# Semiconductor Laser Multi-Spectral Sensing and Imaging

**DOI:** 10.3390/s100100544

**Published:** 2010-01-13

**Authors:** Han Q. Le, Yang Wang

**Affiliations:** 1 Photonic Device and System Lab, Department of Electrical and Computer Engineering, D2-N318, University of Houston, 4800 Calhoun, Houston, TX 77204-4005, USA; 2 Labsphere, Inc. 231 Shaker Street, North Sutton, NH 03260, USA; E-Mail: ywang@labsphere.com

**Keywords:** multispectral, laser sensing, laser imaging, spectral imaging, spectroscopy, chemical detection, semiconductor lasers, mid-infrared lasers

## Abstract

Multi-spectral laser imaging is a technique that can offer a combination of the laser capability of accurate spectral sensing with the desirable features of passive multispectral imaging. The technique can be used for detection, discrimination, and identification of objects by their spectral signature. This article describes and reviews the development and evaluation of semiconductor multi-spectral laser imaging systems. Although the method is certainly not specific to any laser technology, the use of semiconductor lasers is significant with respect to practicality and affordability. More relevantly, semiconductor lasers have their own characteristics; they offer excellent wavelength diversity but usually with modest power. Thus, system design and engineering issues are analyzed for approaches and trade-offs that can make the best use of semiconductor laser capabilities in multispectral imaging. A few systems were developed and the technique was tested and evaluated on a variety of natural and man-made objects. It was shown capable of high spectral resolution imaging which, unlike non-imaging point sensing, allows detecting and discriminating objects of interest even without *a priori* spectroscopic knowledge of the targets. Examples include material and chemical discrimination. It was also shown capable of dealing with the complexity of interpreting diffuse scattered spectral images and produced results that could otherwise be ambiguous with conventional imaging. Examples with glucose and spectral imaging of drug pills were discussed. Lastly, the technique was shown with conventional laser spectroscopy such as wavelength modulation spectroscopy to image a gas (CO). These results suggest the versatility and power of multi-spectral laser imaging, which can be practical with the use of semiconductor lasers.

## 1. Introduction

Optical spectroscopic imaging and the related multi/hyperspectral imaging are highly useful techniques for a wide and diverse range of applications, ranging from microscopic chemical/biological imaging to stand-off mapping of chemical distribution and long-range remote sensing [1–3]. As far as the measurement approach is concerned, the trend has been to use passive multi-/hyperspectral imaging, which employs detectors coupled with wavelength filters/multiplexers to measure the emission or scattered radiation from targets in the natural environment. In some cases, broad-band non-laser light sources are used when illumination is needed.

Lasers uniquely offer radiometric and spectroscopic accuracy and resolution, and multispectral imaging technology can be greatly expanded with the laser. There are applications in which the laser multispectral capability provides invaluable performance; some examples are in the field of LIDAR [4]. For the last few decades since late 1970s to early 1980s, the value of multispectral LIDAR has been well demonstrated as numerous work developed multi-wavelength or tunable/frequency agile LIDARs for applications that range from chemical agent detection [5,6] to atmospheric sensing [4]. Interestingly, the use of multi-wavelength capability is not only for atmospheric gas spectroscopy [7–12] but also for the λ-dependence effect of aerosol scattering [13–17]. More recently, supercontinuum, broadband, or multi-lines LIDAR have also been developed [18–20] for these similar applications.

However, spectral imaging is a more general concept than spectroscopic chemical detection. There is a distinction in the concept. Spectral imaging involves the use of spectral discrimination to segment or classify different objects in an image even without *a priori* spectroscopic knowledge of the objects. In this sense, laser multi-spectral imaging can be viewed as the active counterpart of the passive technique but with laser radiometric accuracy and spectroscopic versatility. Passive spectral sensing must make some estimation on the ambient incident radiation on the target, or the thermal condition of the target *vs.* its ambience, and the background radiation. Laser spectral imaging does not suffer from this uncertainty. Naturally, “spectral” implicitly includes spectroscopy, and laser offers techniques such as Raman, fluorescence, photothermal, photoacoustics, or nonlinear optics that are not available with the passive technique.

Compared with point spectroscopic sensing, the imaging function is essential for certain concepts of operation. Consider for example the case of a small contaminated spot or a speck of substance of interest in a scene that is cluttered with many objects. Point spectroscopic detection can be applied if the suspected spot is known. This means the user must guess roughly where it is, then scans the instrument and searches for it. This scanning is basically a form of “manual” imaging. Automated imaging enables searching for the target rather than just “guessing” and identifying the target.

A practical challenge with laser multispectral imaging is that it is technically difficult and costly to integrate many large laser systems to obtain a wide spectral coverage. Tunable lasers can be used, but it is difficult to obtain a wide tuning range. In addition, the tuning must be fast so that the target does not change much over the tuning period in order to avoid spectral distortion; and complex and expensive frequency-agile tunable lasers are required.

What makes the technique interesting recently is the advance of semiconductor lasers. Semiconductor lasers are small, compact, affordable, available over many spectral regions, and amenable to multi-spectral system integration. Certainly, their power and brightness are somewhat limited, and they are not meant to replace large, powerful lasers in those applications that demand them. But there are also applications that require only modest power, and they truly offer practicality and opportunities to develop the methodology and technique for multispectral laser imaging.

This paper describes some recent studies [22–27] in laser multi-spectral sensing and imaging with semiconductor lasers ranging from near-IR (NIR) to midwave- and longwave-IR (M/LWIR), showing the technique capability and potential for spectroscopic discrimination of objects. The essence of this work is imaging, in the same spirit of passive spectral imaging and is not limited to spectroscopic sensing in the conventional sense of those works mentioned above [4–17]. A recent work also demonstrated the use of multispectral semiconductor laser imaging for stand-off explosives detection using thermoabsorption spectroscopy [28,29], showing the promise of this technique. This paper focuses on two aspects of the technique: the system design issues with the use of semiconductor lasers, and the test and evaluation of the intrinsic capability of laser spectral resolution for spatial discrimination with examples of chemicals and materials.

## 2. Basic Aspects of the Technique

### 2.1. Review of generic concepts

**Figure 1.**
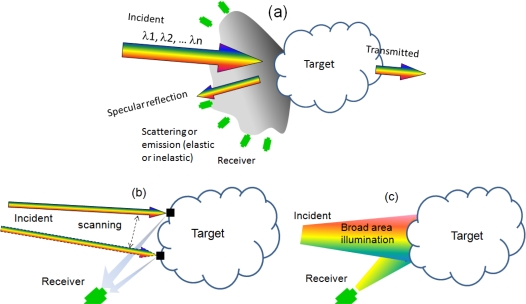
(a) Top: generic concept of multispectral laser imaging. (b) Lower left: imaging by scanning and point-by-point mapping; (c) Lower right: imaging with broad-area staring receiver arrays.

The generic concept of laser multi-spectral imaging is quite simple and is illustrated in Figure 1(a). A multi-spectral laser source excites the target, which can be a gas or condensed matter. The receivers, which can be single-element detectors, arrays, or focal plane arrays, measure the target responses. Being both imaging and spectroscopy, the technique can employ any combination of approaches from either field. Imaging can be achieved by scanning as illustrated in Figure 1(b), where the directionality of the laser beam is used to map point by point, or by staring as illustrated in Figure 1(c), in which the entire illuminated area is mapped. A hybrid approach can be achieved by applying the staring mode over a small illuminated area, and the scanning mode over a large area. All imaging techniques are well established, employed from short-range laser scanners to longer range 3D LIDAR. In addition, other hybrid approaches including spatial encoding or multiplexing techniques, similarly to structured light can also be applied. Which approach to use depends on applications; however, as discussed in Section 3, it is important to consider the system optimization issue for low-power semiconductor lasers, which is more complex than just basic simple noise considerations.

For the spectral measurement of the target, there are several spectroscopic techniques. Most common are absorption, which involves measuring elastic scattering, and fluorescence or Raman scattering, which involves inelastic scattering. In principle, any specific technique can be applied, e.g., WMS (wavelength modulation spectroscopy), nonlinear spectroscopy such as CARS (coherent anti-Stokes Raman scattering), two-photons, and other multi-wave mixings, or non-optical responses such as photoacoustics and thermal radiation (thermoabsorption).

The signal *S*(*λ*;**r**) is a function of wavelength *λ* and position **r**, obtained by scaling the detected signal *P*_scat_(*λ*;**r**) *vs.* excitation laser power, *i.e.*, *S*(*λ*;**r**) = *P*_scat_(*λ*;**r**)/*P*_inc_(*λ*) for linear spectroscopy, and other appropriate scaling can be applied for nonlinear processes. An essential distinction is the priority of the two variables *λ* and **r**. For spectroscopic detection, *λ* is the key variable. A multi-spectral image is a set of spectra ${\left\{S\left(\lambda_{m};r_{p}\right)\right\}}_{m=1}^{L}$ at location **r***_p_*, which is not necessarily the same as a set of intensity images ${\left\{S\left(r_{p}\right);\lambda_{m}\right\}}_{m=1}^{L}$ that is obtained for different *λ*’s. Suppose two intensity images {S(**r**; *λ*_1_)} and {S(**r**; *λ*_2_)} are obtained independently, each can be multiplied by an arbitrary non-zero constant: *A*_1_{*S*(**r**; *λ*_1_)}, *A*_2_{*S*(**r**; *λ*_2_)}, and the integrity of each image is maintained. Yet, {*A*_1_*S*(**r***_p_*; *λ*_1_), *A*_2_*S*(**r***_p_*; *λ*_2_)} does not constitute a valid spectrum of pixel **r***_p_*. An example of such a problem is when various single-*λ* images are taken at different times for which the illumination condition has changed unknown to the system. The result is spectral distortion of each pixel. Thus, it is essential to consider measurement methods that minimize the spectral distortion of ${\left\{S\left(\lambda_{m};r_{p}\right)\right\}}_{m=1}^{L}$.

There are two basic approaches to interpret the spectral signal *S*(*λ*;**r**). The phenomenological approach uses *S*(*λ*;**r**) as a feature for discriminating various objects in the image. The prior-knowledge approach interprets *S*(*λ*;**r**) with pattern recognition algorithms applied to a library of spectra. Thus, if target locations A and B have different *S*(*λ*;**r**), the phenomenological approach would discriminate them as belonging to different objects, without the need to identify what they are. The prior-knowledge approach aims to identify or classify what they are.

**Figure 2.**
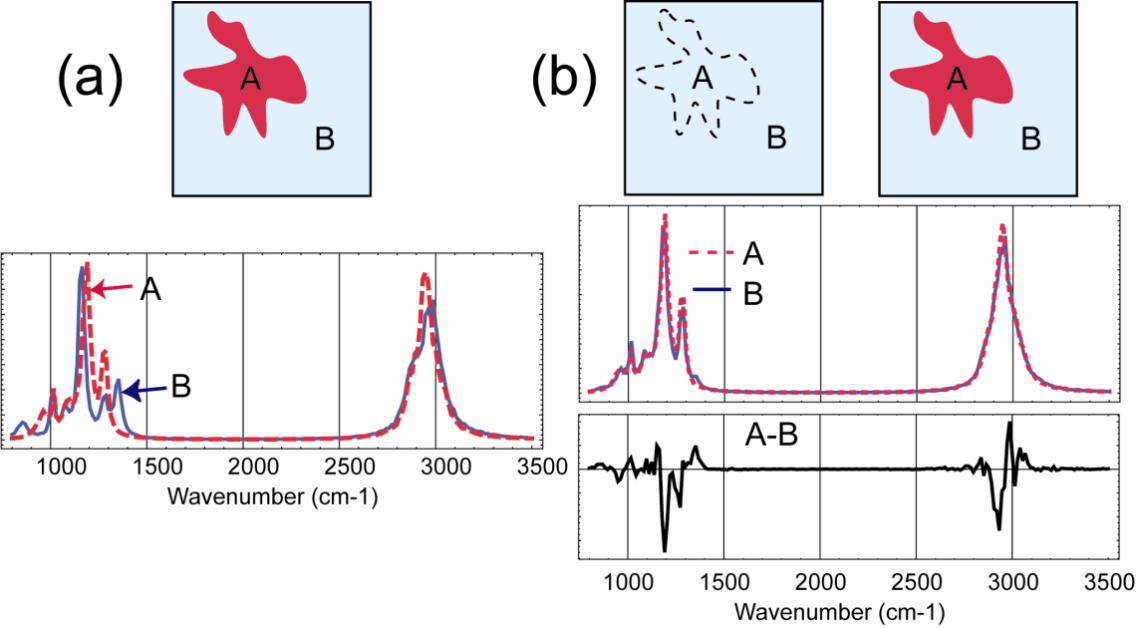
Comparison between absolute spectroscopic imaging and phenomenological imaging algorithm. In case (a), contaminated area A is spectrally distinguishable from B (substrate), and both are spectroscopically identified. The false color image (FCI) of (a)-top shows A and B being distinguishable by both algorithms. In case (b), A and B spectra are so similar that the absolute spectroscopic imaging does not make a distinction, yielding the FCI of (b)-left. However, the phenomenological algorithm detects a statistically significant difference in the A–B spectrum, and hence, can make a distinction in the FCI of (b)-right.

A conceptual comparison of these two approaches is illustrated in Figure 2. Suppose the target is a surface contaminated with some chemical agent. In Figure 2(a), area A and B have spectra as shown. The phenomenological approach can distinguish them based on their difference, and mark them with different colors in a false color image (FCI), even as the approach does not recognize either spectrum. The prior-knowledge approach does not care about their difference (A–B), but tries to match A and B to a library of known spectra. If the matching is successful for both A and B, then this approach is more informative than the phenomenological approach.

However, a key aspect in spectral imaging, as opposed to point spectroscopic sensing, is the spatial discrimination. In some cases, this allows the phenomenological approach to be more informative than the prior-knowledge approach. Consider for example, area A is contaminated with chemical X, but with such a small quantity that it produces only a small signal on top of the much more prominent spectrum of the substrate. Spectra A and B are then very similar to each other, and the prior knowledge approach, when comparing each spectrum independently to the library, may determine that both match to the same library spectrum with, say 95% confidence. Hence, the approach returns a uniform FCI image as in Figure 2(b)-left. Yet, if (A–B) is larger than the measurement uncertainty, the phenomenological approach can make a distinction to produce the FCI as in Figure 2(b)-right. To the user who tries to detect something suspicious, the knowledge that A is somehow different from B is highly valuable. Both methods can be combined, so that the phenomenological approach can make a discrimination to remove the common background between A and B, and yield a difference that represents the contaminant spectrum. Subsequently, this spectrum can be identified by the prior-knowledge method.

The key point is that laser spectral imaging is more than just performing spectroscopic sensing point by point. Imaging offers spatial contrast with the statistics of many-pixel population that allows cluster discrimination in the multi-dimensional spectral space. This cannot be obtained with single-point sensing measurements. In addition, it offers information on target shape and form that can be analyzed in the same vein as that in machine vision to recognize an object. Thus, the combination of spectroscopy, image processing, and pattern recognition enables laser spectral imaging to have a broad application potential.

### 2.2. Issues on spectroscopic interpretation

As laser spectroscopic sensing usually aims to identify the chemical of interest on the first-principle approach, using prior knowledge from a library of spectra, this requires experimental control over the spectral signal *S*(*λ*;**r**) and a theoretical basis for its interpretation. For example, if *S*(*λ*;**r**) is the absorbed transmittance that obeys Beer’s law exp[–*Cα*(*λ*)*L*(**Ω̂**)] through a region with chemical concentration *C*, absorption path length *L*(**Ω̂**) along the laser probe direction **Ω̂**, and *α*(*λ*) is the absorption spectrum, then ln[*S*(*λ*;**r**)] can be matched to the absorption spectra in the database. If *S*(*λ*;**r**) is the Raman or fluorescence spectrum from a rarified medium with no multiple scatterings and no re-absorption, then the spectrum is simply that of the molecules.

However, when imaging an unknown target, it is not always straightforward to interpret *S*(*λ*;**r**). Consider the example in the previous section, the target are spots of chemical agent contaminating on a surface, and *S*(*λ*;**r**) represents diffuse scattering (reflectance), then the signal can be a complicated function of not only the chemical agent dielectric function *ε*(*λ*), but also the film thickness, the laser incident angle, scattering angle, and the substrate spectral property as well as its surface roughness. Examples of this issue are discussed in Section 6. As mentioned, the phenomenological approach can be useful to contrast a contaminated spot *vs.* the area without, but a valid physical model is necessary to extract relevant information for spectroscopic analysis and identification.

The issues of this technique are thus in the ability to control the measurements and the knowledge of target properties. In laser spectroscopic point sensors, all conditions are well controlled to achieve accurate and sensitive detection. Such a condition in general is not always attainable in many applications. The challenge of laser spectral imaging is to optimize the technique to deal with uncontrolled situations, and this is discussed in Section 6.

### 2.3. Issues on measurement methods

At a level more basic than spectral interpretation, the quality of raw data is determined by the SNR (signal-to-noise ratio) of each pixel-wavelength *S*(*λ*;**r**), the spectral fidelity of ${\left\{S\left(\lambda_{i};r\right)\right\}}_{i=1}^{L}$, and the spatial image quality. The first two are most important for spectral identification. System design and measurement methods aim to optimize these figures-of-merit.

An issue is the relative performance of two opposite measurement methods: sequential, which acquires one pixel at a time, and parallel, which acquires all pixels simultaneously, *i.e.*, scanning *vs.* staring. It might appear that the staring approach would be more convenient if the laser power is plenty, and that the scanning approach is preferred when the power is low. But the comparison is not so simplistic; the issue is exactly when a method is more advantageous, and a detailed consideration is crucial for practical applications.

For the sequential method, assume a system that can perform perfect time-division multiplexing, so that at any given time, it can give its total laser power *P* at wavelength *λ* to illuminate only one pixel. Let *NEP* be the average noise equivalent power of the receiver. It is a function of wavelength and other experimental configuration; here *NEP* is taken as a system-averaged Figure. Let *τ* be the measurement time, then the average SNR of each pixel is (using additive Gaussian noise model):

(1) $$\mathrm{SNR}=\rho \frac{P\sqrt{\tau }}{\mathrm{NEP}}$$

where *ρ* is the fraction of incident power that is returned as the signal. From Equation 1, for a given desired SNR, the power required is:

(2) $$P=\frac{1}{\rho }\mathrm{SNR}\frac{\mathrm{NEP}}{\sqrt{\tau }}$$

**Figure 3.**
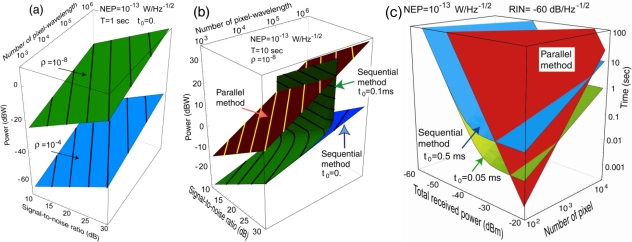
(a) Left: Transmitter power required as a function of pixel-wavelength product number and desired SNR for two return factors *ρ* = 10^−8^ and 10^−4^, with 1-sec total data acquisition time with no dead time between pixel (*t*_0_ = 0). (b) Right: Comparison of power requirement for sequential scanning *vs.* parallel staring method of imaging. Depending on dead time *t*_0_, each method can be best for certain condition. For both, contour lines of 5-dBW apart are also shown. (c) Time needed to acquire a complete multispectral image as a function of received power and number of pixels. For low received power, the sequential scanning method is superior. But the parallel staring method is better with ample signal power. The noise model includes laser relative intensity noise (RIN) as indicated.

A calculation of the power scaling behavior in Equation 2 is illustrated in Figure 3(a). It shows the power requirement as a function of desired SNR and pixel-wavelength product *QL*, with *Q* being the number of pixels and *L* being the number of wavelengths, to acquire the whole image in 1 sec. The two planes correspond to two return factors *ρ* = 10^−8^ and 10^−4^. The former case, *ρ* = 10^−8^ corresponds to very weak return such as in LIDAR; the latter case, *ρ* = 10^−4^ corresponds to short-range scattering. The various lines on the surfaces are power-contours 5-dBW apart, showing the trade-off between SNR and pixel-wavelength product *QL*. The required power for *ρ* = 10^−8^ can be up to 9.6 dBW for *Q* = 128 × 128 and *L* = 50 image with 30-dB SNR. With higher return factor *ρ* = 10^−4^, the lower plane shows that even sub-mW power level (–35 dBW) is sufficient for such an image with 26-dB SNR. Although the calculation is idealistic and does not include other inefficiency and loss, the result shows that over a wide range of conditions from *ρ* = 10^−8^ to 10^−4^, laser multi-spectral imaging is not overly demanding in terms of power, and is within the capability of the semiconductor laser technology for certain circumstances.

To compare the sequential *vs.* the parallel method, it is necessary to consider dead time *t*_0_, which is the time for the scanning system to move from one pixel to another, during which no measurement can be made. Detailed calculation for this comparison is given in the Appendix Section A. The main results can be summarized as follow. Let *T_sequent_* and *T_paral_* denote the net time to acquire an image for a desired SNR and pixel-wavelength product *QL*, then their ratio is [cf. the Appendix Section A, Equation A.9.a)]:

(3) $$\frac{T_{\mathrm{sequent}}}{T_{\mathrm{paral}}}=\frac{\left(1+\eta \right)}{\mathrm{QL}}$$

where, for simplicity, *η* is defined by:

$$\eta \equiv t_{0}/{\left(\frac{1}{\rho P}\mathrm{SNR}\;\mathrm{NEP}\right)}^{2}$$

It appears that the sequential method allows faster (more efficient) image acquisition than the parallel approach for increasing *QL*. Conversely, for the same total image acquisition time *T*, the power required for the sequential approach in Equation (A.2) is less than that for the parallel approach as shown in the Appendix Section A, Equation (A.10):

(4) $$\frac{P_{\mathrm{sequent}}}{P_{\mathrm{paral}}}=\sqrt{\frac{\left(1+\eta \right)}{\mathrm{QL}}}$$

This comparison is illustrated in Figure 3(b), which shows the power requirement for each method as a function of SNR and *QL*. The calculation assumes a weak return, *ρ* = 10^−8^ and a total acquisition time *T* = 10 sec. With zero dead time, the sequential method is certainly more power-efficient, as suggested by the scaling behavior in Equations (3) and (4). Both equations suggest the advantage of the sequential over the parallel method for large *QL*. It is simply the consequence of the additive Gaussian noise model. The upper most plane represents the parallel method, showing that as much as 34.1 dBW is required to achieve the same result as that with 4.6 dBW with the sequential method, represented by the lowest plane with zero dead time *t*_0_. This reflects the ideal case of Equation (4).

However, with realistic dead time and the time constraint on a measurement, the advantage is not for all conditions. With long *t*_0_, such as a switching time between pixels of ∼10^−4^ s, or a wavelength tuning time ∼10^−2^ s, the value of *η* in Equation (3.b) can be large, ∼10^2^–10^4^, which negates the advantage of large *QL*. This is shown by the middle surface in Figure 3(b) that represents the case of *t*_0_ = 0.1 ms. At some point, it curves up rapidly and is no longer advantageous *vs.* the parallel method. The simple reason is that the power must be infinite since there is not enough time left to measure each pixel given the 10-sec time constraint and finite dead time *t*_0_. In practice, hybrid method can be used, for example, all wavelengths can be measured simultaneously to obtain the spectrum of one pixel, and spatial scanning can be applied to the next pixel. Similarly, a small block of spatial pixels can be measured in parallel. This is discussed in the Appendix Section A.

A calculation based on a more realistic noise model is shown in Figure 3(c), which addresses the reverse question of Figure 3(b): given a power *P*, what is the time it takes to obtain an entire image? Figure 3(c) shows the net time *T* as a function of received power *ρP* and the number of spatial pixels *Q*. Here, the calculation assumes that all *L* = 25 wavelengths are measured in parallel, and the spatial pixels are measured sequentially. It employs the hybrid model of Equation (A.8) in the Appendix Section A. As labeled, the top plane corresponds to *T_paral_*. The other two surfaces represent *T_sequent_* with two different dead times *t*_0_ = 0.05 ms and 0.5 ms. The results show the obvious rule that for both methods, the higher the received power is, the faster the measurement will be. When the return power is scarce, the sequential method is better. But when signal power is ample, the parallel approach is faster as expected, as the sequential method is limited by the dead time, unless at large *Q* as shown in Equation (3.a).

A discussion of the model used to calculate Figure 3(c) is given in the Appendix Section A. It involves real system noise behaviors that are more complex than those represented in Equations (3) and (4), and which include laser RIN (relative intensity noise) and the frequency-dependence aspect such as 1/*f*-noise spectral density. The main result is summarized here [cf. Equation (A.14) of the Appendix]:

(5) $$\frac{T_{\mathrm{sequent}}}{T_{\mathrm{paral}}}=\frac{\left(1+\eta \right)}{\mathrm{QL}}\times \frac{{\mathrm{NEP}}^{2}(f_{s})+{\left[\rho P\;\mathrm{RIN}(f_{s})\right]}^{2}}{{\mathrm{NEP}}^{2}(F_{p})+{\left[\rho P\;\mathrm{RIN}(F_{p})/QL\right]}^{2}}$$

In Equation (5), explicit frequency-dependence of the noise is shown, where *f_s_* and *F_p_* represent the measurement frequencies of the serial and parallel methods, respectively, and are given in the Appendix Section A, Equations. (A.13.a,b). Equation (5) shows the complexity in comparing the two methods, which can be very system-dependent and application-specific since different noise terms can dominate in various conditions. In general, since *F_p_* ≪ *f_s_*, the 1/*f*-noise component can be a critical factor in favor of the sequential method, which was indeed observed experimentally in this work.

The key point is that it is necessary to conduct detailed SNR analysis and calculations in order to determine the optimal method for a given circumstance. This system engineering issue is quite relevant to practical applications, which often have constraints or requirements in regard to laser power, collection optics, image resolution, and measurement time. To deliver the best performance possible under these conditions, a system cannot be based on any arbitrary method. Analysis of a nature similar to that for Figure 3(c) is essential.

Beyond the SNR of S(*λ*;**r**), the spectral integrity of ${\left\{S\left(\lambda_{i};r\right)\right\}}_{i=1}^{L}$ is critical. If the target is dynamic, changing its position or properties over the duration of *T_sequent_* or *T_paral_*, there is a risk of spectral and spatial distortion. The nature of the distortion is different for each method, and the parallel method suffers less critical spectral distortion than the sequential method. Thus, measurement method and system optimization cannot be expressed with some rigid rules. Figure 3(c) reflects only a general guideline. The parallel method is usually suitable when there is ample laser power and the image does not require a large number of pixels, and the opposite is true for the sequential method. However, not the least important is the practical issues. For example, large FPA (focal plane array) can be expensive and have the issue of pixel uniformity, while fast scanning technology may require complex control and stabilization in addition to wear-and-tear if using mechanical moving parts. The design and optimization thus must be done for each specific system and application.

## 3. Experimental System

This paper discusses a number of laser spectral imaging studies involving absorption or diffuse reflectance and scattering [22–25]. The focus was not about detecting or investigating some specific chemicals or objects of interest, but to evaluate the methodology, capability and potential of the laser multispectral imaging technique. As mentioned in the introduction, the challenge of broad spectral coverage is usually a key issue. A notable feature is the use of semiconductor lasers, which offer practical and affordable wide spectral coverage by combining many lasers.

### 3.1. System architecture, lasers, and optical hardware

**Figure 4.**
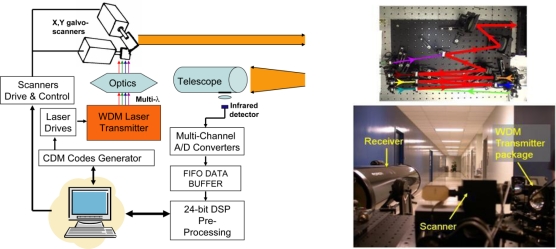
Left: block diagram of the multi-spectral laser imaging system. Right-top: wavelength-division multiplexed transmitter for vis-near-IR diode lasers. Right-bottom: mid-IR system.

The experimental method involves parallel, simultaneous measurements with all wavelengths to acquire the spectrum of a pixel, and sequential scanning to acquire the spatial image. This was done by combining many laser beams into a common aperture, using coarse wavelength-division-multiplexing (WDM) with thin-film bandpass filters as illustrated in Figure 4(a). The block diagram of the system is illustrated in Figure 4(b).

Imaging was achieved by using an X–Y galvanometer scanner to raster-sweep the multi-wavelength beam. The system is laser-power limited, with power ranging from <0 dBm to 10 dBm. Coupled with the return factor *ρ*∼10^−8^ to 10^−4^, the scanning method is most appropriate as discussed above. The WDM approach with simultaneous measurements of all wavelengths is essential to avoid spectral distortion as mentioned. Beam overlap is also crucial to avoid the parallax artifact that can cause spatio-spectral distortion. The beam centroids are overlapped within 1/10 of the beam spot size at their waists, and the beam directions are within 50 μrad of each other.

A key feature is the application of scalable code-division-multiplexing (CDM) architecture for modulation and demodulation to simultaneously measure and distinguish various wavelengths [23–25]. Each laser is modulated with its own unique code. A receiver is capable of receiving and decoding all signals simultaneously. The more wavelengths a system has, the more efficient this approach will be. This architecture is suitable for multi-spectral laser imaging, as opposed to imaging with different laser wavelengths. It is less susceptible to spectral distortion than a method that captures the images sequentially with different wavelengths at different times, as discussed in Section 2.1.

Two semiconductor laser packages were used, a near-IR package with five to seven wavelengths from 0.65 to 1.5 μm, and a mid-IR/long-IR package with four wavelengths from 3.3 to 9.6 μm. The number of wavelengths is modest compared with typical passive multispectral systems, which can have 100 s of wavelengths. However, the goal here is not to perform high resolution spectroscopy but to test and evaluate the essential concept of laser multi-spectral imaging. In fact, the capability and potential of this technique can be demonstrated even with this modest number of wavelengths. The reason for the relatively low number of wavelengths here is not due to some technical limitation but mainly affordability and functionality consideration. Presently, semiconductor lasers in the 0.65–1.5 μm range are highly affordable thanks to the economies of scale of various applications in this wavelength range, but this spectral region is not useful for molecular absorption measurement, being barely in the 3^rd^ overtone bands. More wavelengths are not necessarily useful for the experiments in this work, which did not involve objects with strong color variation in this range. The mid-IR lasers 3–12 μm are spectroscopically more useful, but not as affordable, although they do have the potential to be inexpensive with volume production.

The receivers were simply designed with configurations appropriate for the wavelengths used and the level of scattered light power. The optics include lenses with NA from 0.3 to 0.5, with AR coating for the appropriate spectral range. The receiver aperture diameter ranges from 5 to 10 cm for strong signal conditions. For longer-range and weak signals (M/LWIR standoff measurements), a 12’-parabolic reflector in a converted Cassegrain telescope was used. A variety of thin-film filters were employed as needed. Polarization optics for Stokes parameter measurements were also available for the vis-near-IR setup, but the results [26,27] are not relevant to the results discussed in this paper. The detectors include Si and InGaAs for near-IR, and a combination of InSb and HgCdTe with a bandpass beam-splitter for M/LWIR.

### 3.2. Signal processing and system evaluation

Dedicated home-built electronics include high-bandwidth (10–100 MHz) transimpedance amplifiers (TIA) integrated with appropriate detectors. In addition, a data acquisition board converts the signal with a 12-bit ADC at a rate from 20–200 MS/s, which is subsequently processed with a DSP function to extract the CDM signals. The processed signal is ten acquired with a commercial computer data acquisition system. A key performance feature was the simultaneous measurements of all wavelength signals (on the time scale of one full CDM chip sequence) without cross-talk (<–30 dB), which could also be further filtered out at higher-level signal processing with the computer.

Noises were characterized at every node of the system, and have been discussed elsewhere [24,25]. Laser RIN was minimized by stabilizing the laser driver electronics, including the use of battery to reduce the 1/*f*-component. Detector intrinsic noises were typically only 2–5 dB higher than manufacturers’ specifications. The TIA’s were designed for low noise, and the TIA-ADC combination added a typical noise Figure of only ∼2.5–6 dB, the worst being for the high-bandwidth cases.

However, a further analysis showed that it was not the noise, but the 12-bit ADC that was responsible for a limited dynamic range and a low resolution of the signal amplitude. This translated into a worse spectral resolution for multispectral images. It was calculated that the system could have substantially better performance with 24-bit resolution to fully record the range of backscattered signals. In many cases, weak returned signals that were well above the noise were under-resolved digitally because of more intense specular scattered lights in the same image. Hence, the results reported in the following sections should be viewed with the perspective that they were not yet at the laser-power limit (even as low as the power was) but still limited by the system processing electronics. Nevertheless, all experimental results were obtained at or near the expected system noise level. There were some systemic errors in some cases, but did not affect the results discussed here.

## 4. Experiment Design and Result Overview

The experimental objectives were to test the performance and capability of the system for multi-spectral imaging. The spectroscopy of various targets is not the main interest; the targets were selected to simply represent a variety of common man-made and natural materials. The specific aspects of laser multispectral imaging of interest are:

- The technique intrinsic capability of multi-spectral vector resolution that helps spatial discrimination with examples of chemicals and objects;
- The technique capability to reduce spectroscopic ambiguity, as compared with passive spectral imaging with examples on glucose sensing and on common drug pills imaging;
- And furthermore, to compare its compatibility with conventional spectroscopic sensing, results on wavelength modulation spectroscopic imaging for not only CO gas but other objects in the scene is also described.

For the first aspect, multi-spectral resolution here means the discrimination of normalized spectral vectors ${\left\{S\left(\lambda_{i};r\right)\right\}}_{i=1}^{L}\equiv S$ from each other. It does not mean the resolution of two close spectral lines since the only fixed discrete wavelengths are used here. A key issue in spectral imaging is to distinguish the spectra of two pixels, which are said to be resolvable if their normalized spectra difference is larger than measurement uncertainty:

(6) $$||S_{1}-S_{2}||\geq rM(\Sigma_{1};\Sigma_{2})$$

where ||**S**_1_ – **S**_2_|| represents the distance between them in certain metrics, *M*(**Σ**_1_; **Σ**_2_) is also a metric to measure their variance tensors that represent measurement uncertainty, and *r* is a criterion factor. As a simple example, a metric would be the Mahalanobis distance between the two vectors [36]. A simple example when there is no correlation between various spectral components is:

(7) $$\frac{||S_{1}-S_{2}||}{M(\Sigma_{1};\Sigma_{2})}\equiv \sqrt{\frac{{\left(S_{1\lambda_{1}}-S_{2\lambda_{1}}\right)}^{2}}{\sigma_{1\lambda_{1}}^{2}+\sigma_{2\lambda_{1}}^{2}}+\frac{{\left(S_{1\lambda_{2}}-S_{2\lambda_{2}}\right)}^{2}}{\sigma_{1\lambda_{2}}^{2}+\sigma_{2\lambda_{2}}^{2}}+\cdots \frac{{\left(S_{1\lambda_{L}}-S_{2\lambda_{L}}\right)}^{2}}{\sigma_{1\lambda_{L}}^{2}+\sigma_{2\lambda_{L}}^{2}}}\geq 1$$

where $\sigma_{n\lambda_{m}}^{2}$ represents the total measurement uncertainty that includes any systemic bias and errors. A key value in active laser spectral imaging is the control and knowledge of $\sigma_{n\lambda_{m}}^{2}$, as compared with passive spectral imaging that deals with unknown or insufficient knowledge of the ambient illumination condition. The results in Section 5 indicate that even for low-power short-range standoff system, laser spectral imaging can still perform significant spectral-spatial discrimination of various objects, owing to the low value of $\sigma_{n\lambda_{m}}^{2}$.

The result in Section 6 focuses on another aspect of spectral imaging: the ambiguity and uncertainty in interpreting the spectral results. It is well known that the color of an object may appear differently for different viewpoints and illuminating angles and conditions. Laser allows control of the illumination, and while diffuse-scatter imaging can still have significant uncertainty, the problem can be handled to allow detecting and distinguishing intrinsic spectral features from systemic artifacts. Specific cases to discuss the issue include aqueous glucose measurements and the spectral absorption imaging of particulate matters in some drug pills.

Lastly, laser spectral imaging can certainly be employed as just common spectroscopic sensing. A tunable laser was used to perform conventional wavelength modulation spectroscopic (WMS) imaging of a gas. The key point is not the WMS itself, but the imaging aspect that allows multi functional applications. This is discussed in Section 7.

## 5. Results on Spectral Resolution with Mid-IR Imaging

### 5.1. Mid-IR spectroscopy and multi-spectral resolution

The mid-IR region is interesting for spectroscopic imaging owing to molecular vibration absorption. Both passive and active laser imaging systems have been developed to image chemicals in all forms, from gaseous clouds to liquid and solid matters. As indicated in Section 3.1, a limitation here is the laser power, which was quite modest even for short-range (13–40 m) standoff experiments. A further limitation was the signal dynamic range owing to the low resolution 12-bit ADC as discussed in Section 3.2. Furthermore, only four M/LWIR wavelengths were available, which were not specifically chosen for any spectroscopic advantages. Yet, in spite of these limitations, significant capability of spectral resolution was observed with the system.

**Figure 5.**
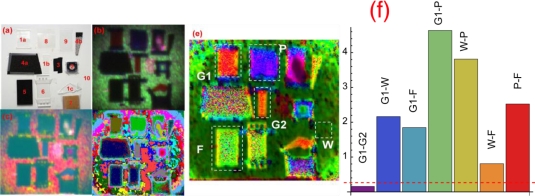
(a) A visible image of the target. (b,c,d) False color images (FCI) from the same IR spectral images with different phenomenological approaches. The algorithms for (b) and (c) do not remove the contrast between the background and the objects, showing that most were highly absorptive (dark appearance) and resulting in under-classification of the objects. The algorithm for (d) over-classifies them and makes more color distinction than physically meaningful. The algorithm for (e) preserves the laser spectral data in lieu of intensity, resulting in physically relevant classifications. Notice that colorless materials (black or transparent) in (a) have “colorful” mid-IR signatures in (e). (f) Bhattacharyya distances between various objects labeled in (e). The red dashed line marks the threshold value for two colors to be considered statistically different. As shown, various objects are spectrally more distinguishable than the FCI in (e) can represent [24].

Figure 5 illustrates the result on a target consisting of pieces of common materials located at 13 m away [24]. It only served as a target for system testing rather than for any specific interests. A photograph of the target is shown in Figure 5(a). From the 4-λ mid-IR spectral images, various phenomenological approaches can be applied to produce the FCI’s in (b–d). The algorithm for the FCIs in Figure 5(b,c) does not remove the contrast between the bright wall background and the absorptive objects, resulting in under-usage of spectral information, since various object spectra that are statistically different are lost in comparison with the bright wall. The algorithm of the FCI in 5(d) over-uses spectral information because it does not take into account noises, and contrast-enhances statistically irresolvable spectra. The FCI in 5(d) is a balance between these two extremes, producing an image with reasonable discrimination among the various objects. Materials that appear only as black or transparent in the visible are clearly distinguishable in the M/LWIR images.

However, the FCI’s in Figure 5 are only for illustration, not for quantitative evaluation of the system capability. For the latter, a key criterion is to consider whether the system is able to discriminate objects in consistence with their spectroscopic signatures.

**Figure 6.**
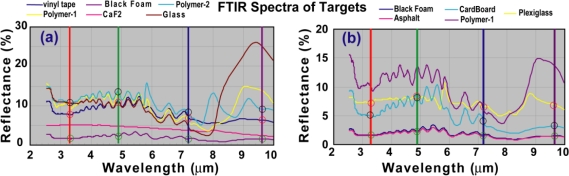
FTIR reflectance spectra of the target materials used in Figure 5. The spectra were calibrated with a gold mirror. The vertical lines mark the laser wavelengths used in spectral imaging. The materials were highly absorptive and systemic background artifact dominates some spectra [24].

For this test, FTIR reflectance spectra of various objects were obtained and shown in Figure 6. They were calibrated against a gold mirror which served as a reference. Most materials were strongly absorptive and their spectra were dominated by systemic background artifacts in the 3–8 μm region, and have some characteristic signatures in the 8–10 μm fingerprint region. The correspondence between the objects in Figure 5(a) and the materials in Figure 6 is as follows: 1a, 1b, 1c: different types of glass and quartz; 2: CaF2; 3: vinyl electrical tape; 4a, 4b: two types of asphalt; 5: black insulator foam; 6: plexiglass; 7: cardboard; 8 and 9: two types of plastic polymer; 10: painted wall. The vertical lines mark the laser wavelengths. One can construct the equivalent 4-λ signatures of the objects from the FTIR spectra, and the anticipated spectral contrast (or distance) between objects can be calculated with criteria in Equations (6) and (7) by scaling for comparable signal amplitude equivalent noises.

The laser system outperformed the FTIR-based criterion. A simple reason was that the FTIR signals from many materials were insufficient to provide any significant spectral contrast. The weak spectral signals, if any were dominated by a large systemic background in the 3–8 μm region. The common systemic background could be verified with the strong correlation function among them. In fact, several materials have practically identical 4-λ FTIR signatures, simply for the lack of sufficient reflectance signal power, such as the black vinyl tape, some polymers, asphalts, and foams. This is the reason for various objects to appear dark black in Figure 5(b,c). Yet, with the laser measurements, the object spectra were statistically distinguishable once normalized. For example, the black vinyl tape and a polymer 4-λ spectra form clusters in the 4-D wavelength space that are resolvable. They would not have been distinguishable based on their 4-λ FTIR signatures. This simply owes to the fact that the lasers had sufficient power to generate spectroscopically meaningful backscattered signals from these materials.

A useful statistical metric for the spectral contrast among the materials is the Bhattacharyya measure (or distance) [36]:

(8) $$D_{B}(U,V)=-\text{ln}\int \sqrt{P[S(U)]P[S(V)]}{ds}_{\lambda_{1}}{ds}_{\lambda_{2}}\ldots {ds}_{\lambda_{L}}$$

Where *P*[**S**(**U**)], *P*[**S**(**V**)] are the probability density function of normalized spectral vector **S** of region **U** and **V** that consist of all pixels of the same materials. Larger Bhattacharya distance *D*_B_(**U**,**V**) means larger color difference between two objects. Some results are illustrated in Figure 5(f). They confirm that the system of these 4 wavelengths can distinguish these test materials as well as they should be, including some high absorptive materials with SNR as low as a few dB. Statistically, the materials are more distinguishable with the Bhattacharyya metric than what can be seen from the FCI, which is limited to three colors RGB as opposed to 4-λ data. A simple empirical criterion to test is to randomly divide a pixel population of the same object into two sets and measure their *D*_B_(**U_1_**,**U_2_**). Several such exercises were performed to yield a distribution of *D*_B_(**U_1_**,**U_2_**). Ideally, it should be applied to a population with high SNR for all wavelengths. Unfortunately, there was no such a population. A few clusters were selected and yielded result ranging from 0.05 to 0.32 for very noisy pixels. An empirical mean is shown as the dashed line in Figure 5(f). Indeed, it shows that every object as indicated was distinguishable except for two pieces of glasses, which should indistinguishable as expected.

Another explanation of the laser system ability to outperform the FTIR-based results is the statistics of population. Laser measurements include many sampled points of the materials, whereas the FTIR results came from a single measurement over a spot of the sample (although a larger spot than the laser beam), and hence, they lack the statistics of population. The sufficient data population enables the *D*_B_(**U**,**V**) measurement in Equation (16) to yield reasonable resolution among closely clustered spectral populations.

Thus the result here essentially validates the performance of the laser multispectral imaging system, which met the criterion of spectral discrimination of various test objects. It should be noted that if the FTIR fingerprint region data were used, many materials would also be very well resolved from each other. However, the scope of this test is not about optimal spectroscopic wavelength range. Given the available laser wavelengths, the test could only be applied as it was. In fact, the system capability would have been more pronounced if materials with unique signatures over these four wavelengths had been specially selected. More generally, there is no doubt that passive technique such as FTIR offer the advantage of broad spectral coverage that is a challenge for the laser-based system. Precisely for this reason, as multispectral laser systems acquire more wavelengths, they can be expected to offer the combined advantages of broad spectral coverage as proven with the FTIR passive method, and the laser radiometric accuracy and dynamic range as demonstrated in these test results.

### 5.2. Example of chemical discrimination

**Figure 7.**
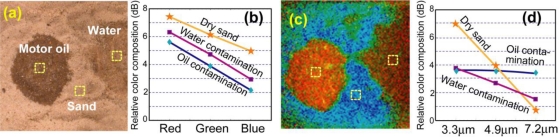
(a) Visible image of sand contaminated with oil and water. (b) RGB colorimetric decompositions of spots marked by dashed yellow square boxes in (a), which shows that the appearance difference between the three marked spots is not spectral (color) but only of intensity (lightness). (c) The M/LWIR multi-spectral false color image makes clear spectral discrimination and not just intensity discrimination between the spots. The reason is shown in (d): they have distinctive M/LWIR spectra.

Figure 7 shows dry sand with patches of oil and water contamination. For the visible image in Figure 7(a), the contamination appears as dark patches, but the distinction is based on intensity, not color as the relative RGB decompositions in Figure 7(b) of the three marked spots appear nearly the same. Their IR spectra in Figure 7(d) are truly different, which reflect in the FCI Figure 7(c), suggesting different chemicals.

**Figure 8.**
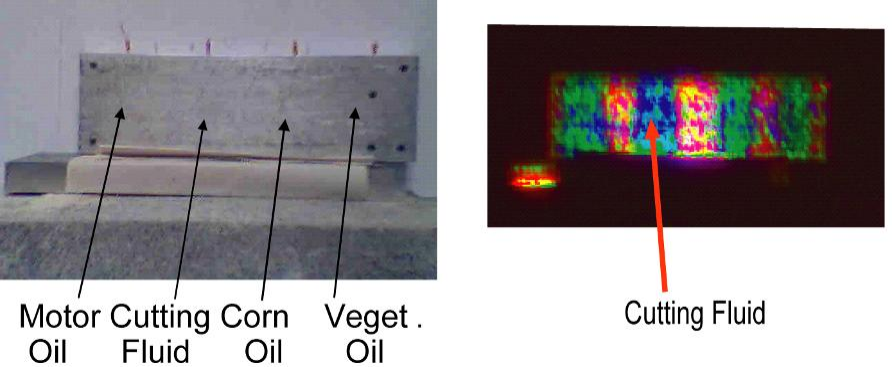
Left: visible image of an aluminum plate contaminated with 4 thin-film stripes of oils. Right: the multi-spectral MIR false-color image showing the oil films as green/blue and the metal as red/yellow. Petrochemical cutting fluid displays a bluish hue that is statistically distinguishable from the organic oils.

Figure 8(a) shows the visible image of an aluminum plate contaminated with four different oils, two are bio-organic and two are petroleum hydrocarbons. The oil films were estimated to be <100 μm thick. In the IR multi-spectral FCI of Figure 8(b), the oil patches appear as green/blue, and the metal appears red/yellow. The Al plate had strong specular and speckles components, overwhelming the receiver dynamic range. Attenuating the optical signal to avoid saturation by the Al signals rendered all other features noisy. This is the problem of limited dynamic range as discussed in Section 3. Nevertheless, this case is also an example of the discussion in Section 2.1 about discrimination without spectroscopic identification.

The mid-IR signatures of all oils with four wavelengths were similar and not sufficient to identify individually, although all were distinguishable from bare Al. Spectral discrimination shows only cutting fluid oil as being slightly different from the others. Measurement at just one point would not have been sufficient to infer the difference between the cutting fluid from the others with high confidence, given the signal-to-noise level of the data. Yet, imaging with phenomenological spectral contrast yields sufficient statistics with its Bhattacharyya distance 1.03 from the others, allowing inference with higher confidence that the area was indeed contaminated with something different from the rest.

**Figure 9.**
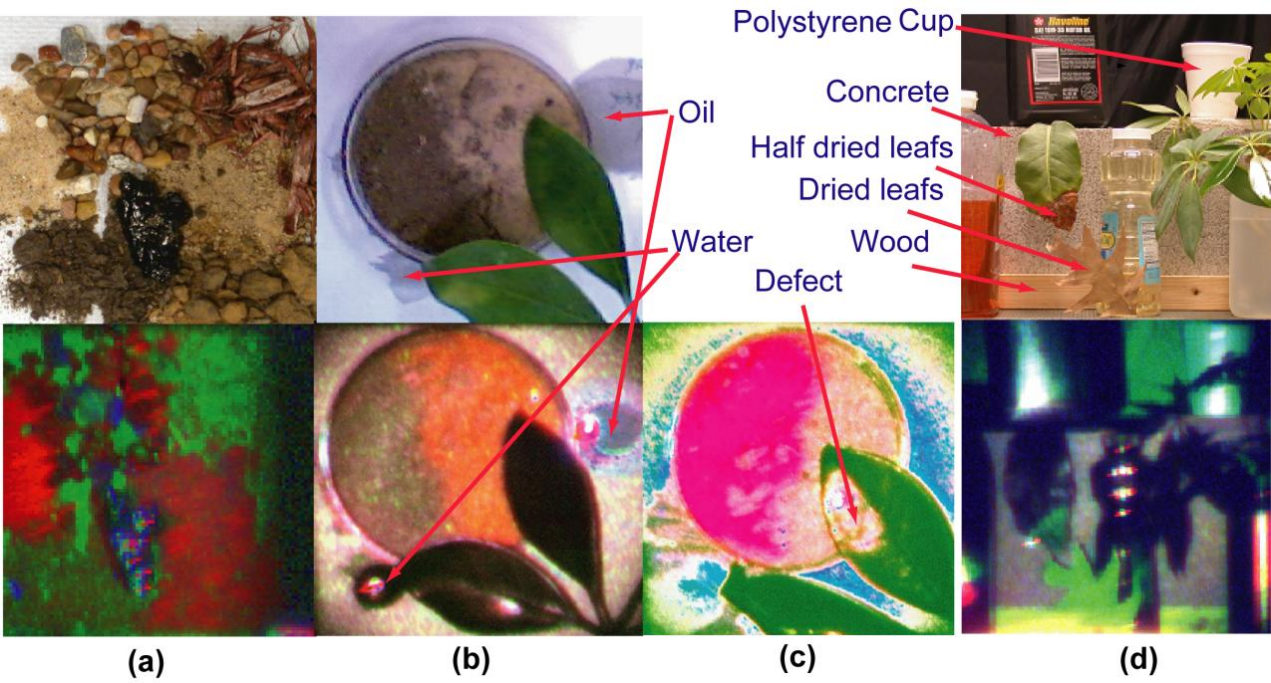
*Top row:* target photographs. *Bottom row:* corresponding FCI from M/LWIR multispectral images. (a) Mineral collection. The M/LWIR FCI shows sand (quartz) as red, humus soil and woods as brownish/dark green, and asphalts as bluish. The beam was ∼2.5 cm, and larger than most pebbles. (b) Sandy soil, humus soil, and leaves. The M/LWIR FCI shows sandy and humus soils have different colors. (c) M/LWIR FCI of the same target in (b) under a slightly different arrangement. A barely discernible yellowish spot of a leave became very pronounced in the FCI. (d) Household objects. Dried leaves are distinctive from green leaves (black because of weak signals). A piece of wood appears as yellow; and concrete appears as gray. Other shiny objects with specular reflection caused the dynamic range problem as spectra of weak signals (dark region) were lost in signal digitization (flare problem).

Various images of other natural objects are shown in Figure 9. The top row shows the target photographs. The bottom row shows their FCI from the M/LWIR multispectral images. Figure 9(a) shows a soil collection; the FCI shows distinction among various types. Figure 9(b,c) show sand, humus soil, and leaves. The FCI in Figure 9(c) shows that a part of a leave that barely appears yellowish in the visible becomes pronounced in the IR. The fact that Figure 9(b,c) FCIs reveal different features of the same target is simply because the 4-λ images contain more spectral resolution than what can be projected into 3-λ RGB FCIs for human perception. Thus, Figure 9(b,c) FCIs represent two different 4D-to-3D projections that show different distinction. In Figure 9(d) FCI, dried leaves appear as light green, compared with black for green leaves. The problem of Fig, 9(d) was also the limited dynamic range of the system as the strong specular reflection caused the system to reduce the sensitivity to other objects, rendering them with insufficient resolution for spectral discrimination.

## 6. Results on Diffuse Scatter Imaging with Near-IR

### 6.1. The spectral issue of diffuse scattering

Several images in Section 5 show spectral variation within a homogeneous object. This variation can be attributed to signal noises. However, even without the noises, there is an intrinsic spectral variation effect due to the scattering process that is a function of the viewing angle, the illumination condition, and the random surface structure of an object. This is the reason why a homogeneous object may appear to have spatially varying hue. A challenge in multispectral imaging is to distinguish this type of variation from that associated with the material dielectric property. This section considers this issue.

**Figure 10.**
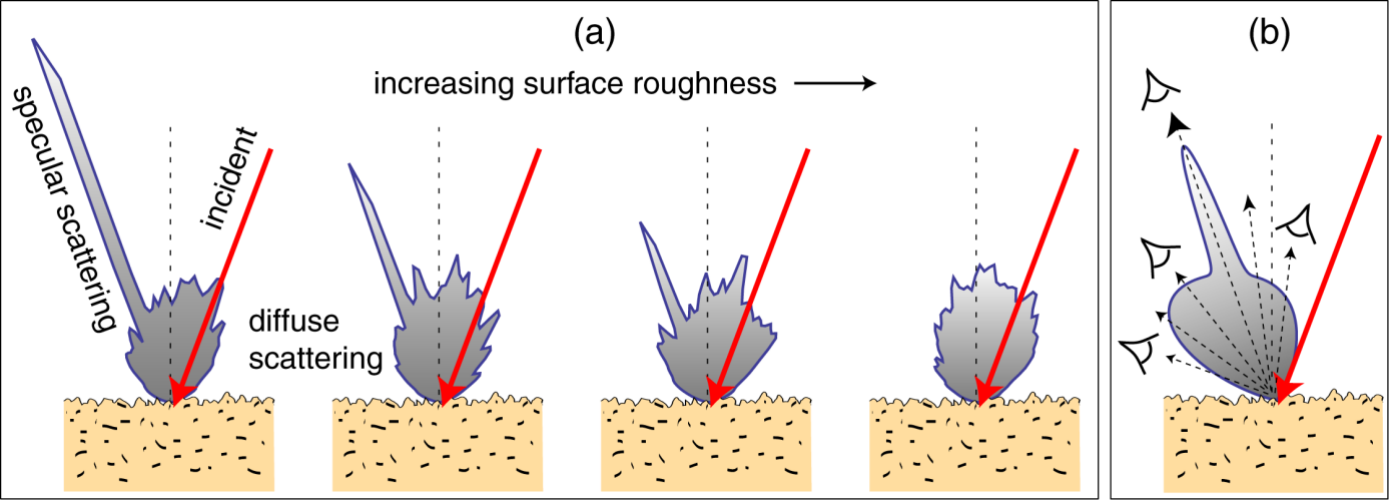
(a) FDTD calculation of scattered light from a random surface with roughness increasing from left to right. (b) A phenomenological Cook-Torrance scattering model.

Figure 10 illustrates the scattering that ranges from strongly specular to highly diffused from a random surface, which ranges from smooth to rough from left to right. The calculation was based on the FDTD (finite difference time domain) method. The surface is statistically homogeneous in the sense that they were generated with a statistical model that assumes a surface distribution with unique characteristic length and surface roughness. Real surfaces are much more complex and the issue will be discussed in Section 6.3. For comparison, a model based on the Cook-Torrance bidirectional reflection distribution function (BRDF) with two surface parameters is plotted in Figure 10(b). The difference between the two calculations is that the FDTD does not make distinction of the specular and the diffused, as the result is from numerical solution of the wave equation, whereas the BRDF involves phenomenological incoherent summation of two distributions.

The issue is illustrated in Figure 10(b). As a function of the viewing angle, the observer (represented by the eye) will see different color from the object. When the observer looks at highly-diffuse scattered light, the color will be somewhat dominated by the surface absorption property, determined by Im*ε*(*λ*;**r**). When the observer looks at specular-reflection-like scattered light, the color will be somewhat dominated by the surface Fresnel reflectance, determined by Re*ε*(*λ*;**r**). This effect is not only a function of the viewing angle, but also of the illumination angle and especially the surface microscopic structure, morphology and subsurface bulk structure. This problem raises the challenge of interpreting spectroscopic information in multi-spectral images.

The interest of spectral imaging is not in the scattering angular distribution, but to infer the substrate dielectric *ε*(*λ*;**r**) spectroscopic properties from the scattered light. The latter can be described in terms of the differential scattering coefficient:

(9) $$S({\hat{\Omega }}_{I};{\hat{\Omega }}_{S})\equiv \frac{P({\hat{\Omega }}_{I};{\hat{\Omega }}_{S})}{P_{\text{inc}}}$$

where *P*(**Ω̂***_I_*;**Ω̂***_S_*) is the scattered power per sterad, **Ω̂***_I_* is the incident direction, and *P*_inc_ is the incident power. All three quantities **S**(**Ω̂***_I_*;**Ω̂***_S_*), *P*(**Ω̂***_I_*;**Ω̂***_S_*), and *P*_inc_ are implicitly λ-dependent, but variable λ is omitted for simplicity. As illustrated in Figure 10(a), the issue is that for a random surface, there is no simple relationship between **S**(**Ω̂***_I_*;**Ω̂***_S_*) spectrum and *ε*(*λ*;**r**). Only for a smooth surface, in which **S**(**Ω̂***_I_*;**Ω̂***_S_*) is the Fresnel reflectance is there a known analytic relation between **S**(**Ω̂***_I_*;**Ω̂***_S_*) and *ε*(*λ*;**r**).

The function **S**(**Ω̂***_I_*;**Ω̂***_S_*) is conceptually similar to BRDF, or more generally BSDF (bidirectional scattering distribution function). But a key difference between **S**(**Ω̂***_I_*;**Ω̂***_S_*) here and the common BSDFs often used in computer graphics is that the latter are phenomenological models; some are based on ray optics and thus do account for the field coherent effects (interference, diffraction) that can be significant in spectroscopic measurements. In computer graphics, the light is often phenomenologically approximated as a linear combination of reflection and absorption, which can be acceptable to the human visual experience but is not optically correct for spectral sensing.

When an object has very pronounced characteristic spectroscopic features, the above effect might not appear important. An object with a pronounced color can easily be recognized under almost any illumination condition and viewing angle. But when trying to compare two “hues” with subtle differences, such as detecting some small contamination, this effect becomes important. An intuitive example is when we humans must distinguish two similar hues, such as two close shades of paint. We often tilt and rotate the objects to look at different angles, and/or change the illumination in order to find a favorable condition that can enhance their spectral contrast to our eyes. Laser measurements offer their advantages in such cases. The next section discusses some experimental results and a theoretical basis for complex diffuse scattering with implication on spectral imaging. The experimental results include the detection of aqueous glucose and contrast imaging of common drug pills.

### 6.2. The case of aqueous glucose

**Figure 11.**
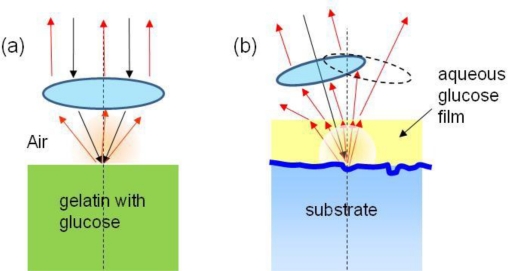
Experimental configurations to measure glucose. (a) glucose was dissolved in the gelatin with a smooth surface. (b) glucose was dissolved in a thin water film or in the substrate with a random surface. For strong diffuse scattering, the optical measurement configuration was not fixed, but varied to study different components of scattered light.

**Figure 12.**
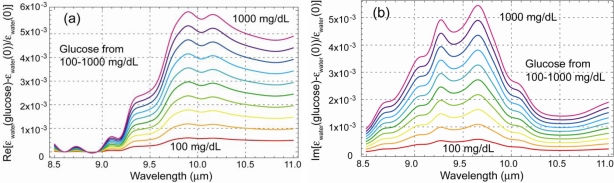
(a) and (b): The Re and Im part of the glucose-induced change of water dielectric constant as a function of glucose concentration, relative to that of pure water.

Prior to theoretical consideration, consider the experimental results on glucose that illustrate the effects of spectral variation discussed above. Although the work [30,31] did not involve imaging, the result is quite relevant and useful not only for considering the complex aspects of spectroscopic sensing but also approaches for optimization. Figure 11(a,b) show the experimental configurations to measure glucose, either from a substrate, or in a thin water film on a substrate that may or may not contain glucose. This problem can be relevant to the detection of any thin film material absorption. The typical glucose concentration in these experiments was from 200 to 1,000 mg/dL (except for one result at 4,000 mg/dL). In the 8–11 μm spectral range, the glucose modification to the water dielectric function is ∼ a few times 10^−3^ as shown in a model calculation in Figure 12 for the Re and Im part of the dielectric constant. The challenge was to detect this small difference of *ε*(*λ*), or in other words, to detect small “hue” variation.

It was found that the backscattered spectrum indeed exhibited spectral variation depending on the incident light configuration, the scattered light collection, and the substrate properties. The spectrum variation can be conveniently (but inaccurately) referred to as Fresnel-like, absorption-like, or neither.

**Figure 13.**
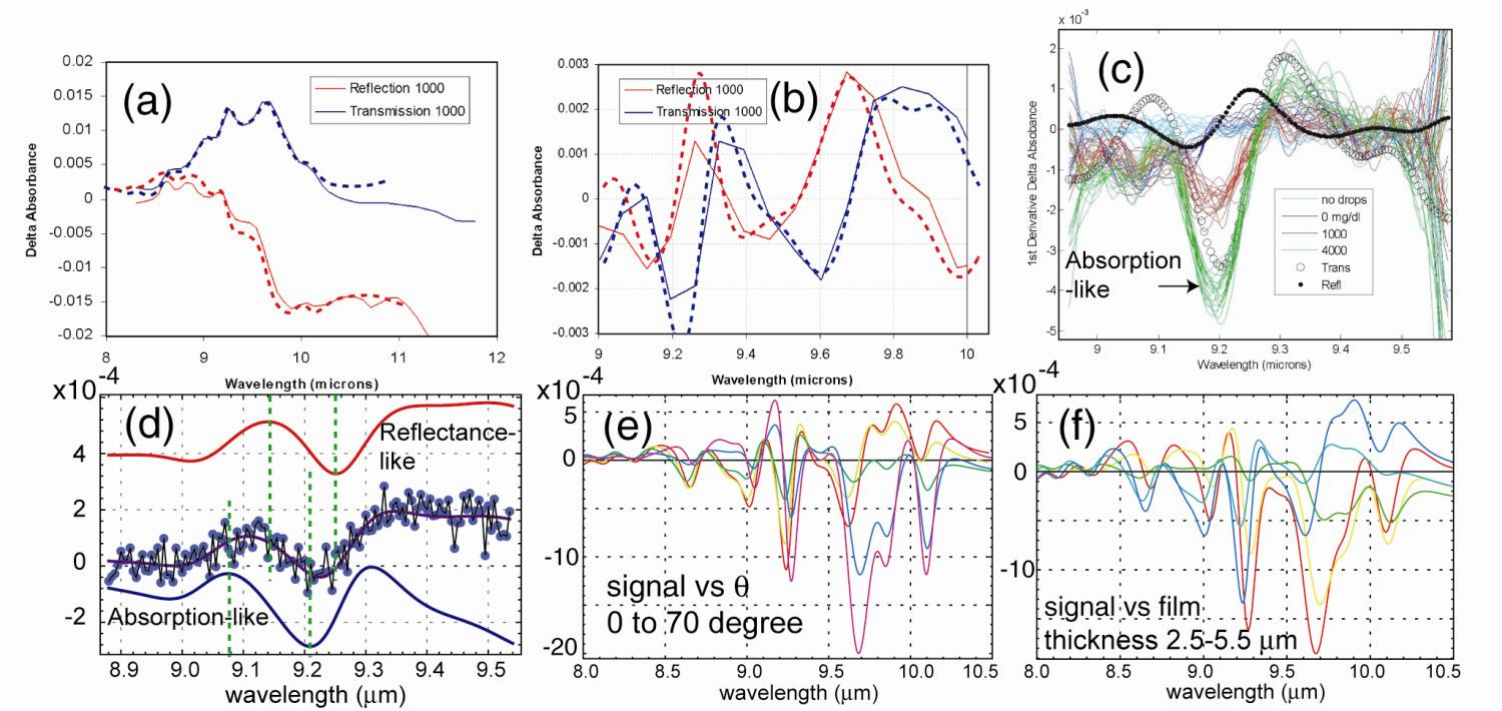
Spectra of aqueous glucose from Refs. [30,31] (a) Reflectance spectrum from gelatin glucose and transmission spectrum of aqueous glucose with modeling (dashed line) (b) Derivative *vs.* wavenumber of the spectra in (a); solid lines are experiments, dashed lines are modeling results. (c) and (d): Derivative spectra of diffuse backscattered signal of aqueous glucose thin film under the experimental configuration in Figure 11(b). In (c), the glucose is in a thin tear layer on a human eye conjunctiva, showing absorption-like result. But (d) is a different result that is neither reflectance-like nor absorption-like, it is fitted with a computer simulation (solid curve fit). (e) and (f): exact calculation of signal in Equation (10), showing significant spectrum variation *vs.* incident angle and film thickness.

Figure 13(a) shows the reflectance from a thick gelatin glucose sample, which is a Fresnel-like spectrum in the 8–10 μm range [26]. For reference, the absorption-dominated result from a transmission cell measurement was also given. Both were obtained by subtracting the measured spectra by that of pure water. The result can be interpreted that the backscattered light was principally from the smooth air-gelatin interface reflection and determined by Fresnel reflection. The dashed curves are results from the computation model showing agreement for this trivial case. Angular-dependence spectral change was also observed [26] as expected.

Figure 13(b) shows the derivative spectra *vs.* wave number, which are more effective to enhance the glucose absorption features in the 9-μm range. The modeling results (dashed curve) also account well for the measurements. Both results of Figure 13(a,b) can be considered as a case of well controlled scattering process, in which there is little ambiguity in the measurement configuration and the nature of the spectrum.

However, more significant are the results of uncontrolled cases, similarly to the detection of contaminants in an uncontrolled, unknown condition. The results in Figure 13(c,d) correspond to the experimental configuration in Figure 11(b), which involves scattering from a thin film of aqueous glucose on a substrate with random surface. The results are less clear-cut about the nature of the signal. Figure 13(c) appears to be “absorption-like”, based on the 4,000 mg/dL result, which can be interpreted as being dominated by the absorption of the thin water film as the incident light made roundtrip through the film before being backscattered from the substrate. Figure 13(d) is different, which although noisy, does not appear to be consistent with the simple interpretation of being either absorption-like or reflectance-like. The film thickness and substrate were different and unknown for both measurements in Figure 13(c,d). A computer simulation of random scattering from the substrate produced a best match for the result in Figure 13(d), which is shown as the solid curve. Both results in Figure 13(c,d) are clear evidence that there are inevitable spectral variations *vs.* scattering configuration and substrate properties. In addition, there was not an algorithm that would allow unique, unequivocal inference of glucose concentration from the scattered light spectrum, since many simulation scenarios could produce similar results within the 8.9–9.6 μm range.

The theoretical implication on detection will be discussed more generally in Section 6.3. Here, some aspects of the problem can be understood by considering the calculation in Figure 13(e,f), which shows the exact analytic result based on the case of a smooth substrate surface and the film is parallel with a uniform thickness. The backscattered signal in this case is the electric field reflectance [cf. the Appendix Section B, Equation (A.18.a)]:

(10) $$r(\lambda ;k)=\frac{\left(k_{a}-k_{f})(k_{f}+k_{s})+e^{2{\mathrm{idk}}_{f}}\;(k_{a}+k_{f})(k_{f}-k_{s}\right)}{\left(k_{a}+k_{f})(k_{f}+k_{s})+e^{{2\mathrm{idk}}_{f}}\;(k_{a}-k_{f})(k_{f}-k_{s}\right)}$$

where: *k_a_* = *k*_0_ cos(*θ*_inc_); $k_{f}=k_{0}\sqrt{\varepsilon_{f}\;\left(\lambda \right)-{\text{sin}}^{2}\;\left(\theta_{\text{inc}}\right)}$; $k_{s}=k_{0}\sqrt{\varepsilon_{s}\left(\lambda \right)-{\text{sin}}^{2}\left(\theta_{\text{inc}}\right)}$; *k*_0_ ≡ 2*π*/*λ*; and *ε_f_*(*λ*) is the dielectric function of the aqueous glucose film with thickness *d*. The calculation is also for the derivative spectrum and the relative change. As shown, the spectral variation is quite substantial as a function of the incident angle [Figure 13(e)] and film thickness [Figure 13(f)]. For a focused beam, the net reflectance (or backscattered) signal detected is [cf. the Appendix Section B, Equation (A.24)]:

(11) $$S({\hat{\Omega }}_{S})\equiv R_{T}\;(\lambda )={\left|\iint_{\begin{array}{l} \text{Receiver}\\ \text{aperture} \end{array}}H({\hat{\Omega }}_{S})d{\hat{\Omega }}_{S}r(\lambda ;k)A(k)e^{i\phi (k)}dk\right|}^{2}$$

where *r*(*λ*;**k**) is given by Equation (10), *A*(**k**)*e^i^^ϕ^*^(^**^k^**^)^ is the amplitude-phase product of the incident beam such that *E*(**r**) = ∫*A*(**k**)*e^iϕ^*^(^**^k^**^)^*e^i^***^k^**^·^**^r^***d***k** is the incident beam electric field, *H*(**Ω̂***_S_*) is the receiver collection efficiency and the integral is not for all **k**, but only those in the direction collected by the receiver aperture. Equations (10) and (11) show the important of the phase of the light. Phenomenological ray-optics approach such as those in computer graphic BSDF would not produce the spectral variation in Figure 13(e,f). However, although inaccurate, it is convenient to think of *S*(**Ω̂***_S_*) as a combination of many rays, some are Fresnel-like specular reflection and some are diffuse-scattered rays from the substrate after being absorbed by the film. Thus, for a random surface that may have heterogeneous morphology and capillary film-thickness variation, any combination of the results of Figure 13(e,f) can be the case. It is not surprising to observe spectral variation under various measurement conditions.

Nevertheless, the essential result is that a deviation of ∼ few times 10^−3^ of *ε_f_*(*λ*) [Figure 12(a,b)] can be detected under uncontrolled (unknown) light scattering condition. The issue here is not that it is difficult to detect a contaminant, but only to determine the contaminant quantity from the scattered light. The specific issue for the glucose experiments was that its concentration could not be determined with the desired accuracy and error limit. The reason is the complexity of the random scattering process. In contrast, under a well controlled configuration without random scattering, similar experiments allowed far more precise glucose measurement with lower concentration [32].

On the issue of sensitivity, part of the problem was also the SNR. The use of derivative spectrum is to bring out the glucose feature from the background. However, those obtained in Figure 13 were not truly wavelength modulation spectroscopy measurements (WMS). They were obtained numerically from λ-tuning spectra, which were obtained at slow speeds (from 1 to 10’s of seconds tuning time), and hence suffered significant 1/*f*-noise. At least 5–10 dB SNR improvement can be expected with high-frequency WMS. In addition, the sample actually changed over the scanning period, including the movement of the substrate, or the slow evaporation of the water film, or the continuing capillary action on a surface. This is also related to the discussion in Section 1 about the need for fast wavelength tuning, or frequency agile capability. The net result was large errors and noises that seemed to limit the sensitivity to ∼ few times 10^−3^ of *ε_f_*(*λ*); otherwise, there was sufficient laser power for detecting <10^−4^ change of *ε_f_*(*λ*).

The implication for spectral imaging is that in spite of the intrinsic uncertainty with random surface diffuse scattering, detection and image discrimination are possible but will require approaches to optimize the measurements and minimize the uncertainty. This is discussed in the next section, which is generally applicable to other problems of a similar nature.

### 6.3. Generalization for diffuse scattering

The basis for detecting a substance via its absorption in random diffuse scattering can be formulated as follow. Here, only elastic scattering is considered, as inelastic scatterings such as fluorescence or Raman have signals of a different nature. One can generally assume that the scattered electric field amplitude **E***_S_* is an unknown but deterministic function of the substrate dielectric function *ε_s_*(*λ*) and the film dielectric function *ε_f_*(*λ;C*_X_), which is dependent on contaminant concentration *C*_X_. The scattered electric field **E***_S_*[*λ;ε_s_*(*λ*), *ε_f_*(*λ;C*_X_)] can be expanded with the first-order Taylor’s series:

(12) $$E_{S}\;\left[\lambda ;\varepsilon_{s}\;(\lambda ),\varepsilon_{f}\;(\lambda ;C_{X})\right]\approx E_{S}\left[\lambda ;\varepsilon_{s}\;(\lambda ),\varepsilon_{f}\;(\lambda ;0)\right]+C_{X}\;\frac{\partial E_{S}}{\partial \varepsilon_{f}}\;{\tilde{\varepsilon }}_{X}$$

where *ε̃*_X_ ≡ *ε̂*_X_ – *με_f_*, *ε̂*_X_ is the specific dielectric function per unit of substance concentration *C*_X_, and *μ* is a substitution factor to account for the displacement of solvent molecules by those of substance *X*. There is of course a dependence on the incident and detection angle **Ω̂**_inc_, **Ω̂**_rec_, but these are omitted for clarity. It should be noted that the model of Equation (12) is generic and not limited to the configuration in Figure 11. In the plane-wave decomposition approach, the incident field is a linear combination of plane wave with wave vector **k**. Each **E***_S_* of Equation (12) then corresponds to the **k**-plane wave component. The detected scattered field is a sum of all **k’s** of those in Equation (12).

Details of the rest of discussion are given in the Appendix Section C, and the key results are summarized here. It can be shown that the scattered light intensity *S*(*λ*) is a linear combination both Re*ε̃*_X_ and Im*ε̃*_X_; and the derivative *dS*(*λ*)/*dλ* additionally contains *d* Re*ε̃*_X_/d*λ* and *d* Im*ε̃*_X_/*dλ*:

(13) $$\frac{d}{d\lambda }S(\lambda )=\frac{dS_{0}}{d\lambda }+2C_{X}\left(\frac{\mathrm{da}}{d\lambda }\text{Re}{\tilde{\varepsilon }}_{X}+\frac{\mathrm{db}}{d\lambda }\text{Im}{\tilde{\varepsilon }}_{X}+a\frac{d\text{Re}{\tilde{\varepsilon }}_{X}}{d\lambda }+b\frac{d\text{Im}{\tilde{\varepsilon }}_{X}}{d\lambda }\right)$$

where *a*(*λ*) and *b*(*λ*) are coefficient functions defined in the Appendix Section C, Equations (A.28). If *a*(*λ*) and *b*(*λ*) are known, the contaminant concentration *C*_X_ can be inferred, given *ε̂*_X_(*λ*) of the substance of interest is known. For a known geometry, *a*(*λ*) and *b*(*λ*) can be computed. However, as discussed in the Appendix Section C, the challenge is that *a*(*λ*) and *b*(*λ*) are not precisely known for unknown substrate surface characteristics. Nevertheless, if the surface statistical properties are known, computer simulation similar to that shown in Figure 10(a) can establish some estimates for their magnitudes. If some reasonable bounds of their values can be assumed, it is possible to estimate a range of magnitude for *C*_X_, although its precise value cannot be determined.

This is the basis of how glucose was detected in the experiments in Section 6.2 above. In particular, it appears that *a*’(*λ*), and *b*’(*λ*) are quite small for these experiments and, this explains the presence of both *d* Re*ε̃*_X_/d*λ* and *d* Im*ε̃*_X_/*dλ* in several spectra depending on the experimental condition. The glucose results and this theoretical consideration entail a number of implications on the strategy of detection of contaminants and interpretation of spectral images. The details are given in the Appendix Section C; the key points are summarized in the follow:

- It is not a rational strategy to search for a fixed spectral pattern, since the relative magnitudes of coefficients *a*(*λ*), *b*(*λ*), *a*’(*λ*), and *b*’(*λ*) are likely to vary substantially *vs.* angles and collection configuration. A flexible spectral pattern matching based on various combinations of the basis functions of *ε_f_*(*λ*) and its derivatives is a more appropriate strategy.
- As a corollary, it is important in spectral imaging not to make arbitrary distinction between two objects of identical nature just because they appear to have different spectra as a result of the measurement conditions as mentioned in (*i*). This is the difference between physics-based processing of spectral images and purely phenomenological image processing.
- Obviously, choosing a spectral range such that *d* Re*ε̃*_X_/*dλ* and *d* Im*ε̃*_X_/*dλ* have unique, special features is essential to detect the contaminant. Higher order derivatives are theoretically even better if there is sufficient SNR.
- It is desirable that coefficients *a*(*λ*), *b*(*λ*), *a*’(*λ*), *b*’(*λ*) of Equation (13) be as large as possible. For spectral imaging, it is desirable to diversify the illumination and detection configuration to search for optimal illumination and viewing angles. This is actually the reason why we humans intuitively tilt and rotate objects to enhance hue contrast as mentioned above. It is also the reason for the large angular variation in Figure 13(e,f) as these coefficients increase for larger scattered angle in this particular case. Not only the amplitude but the phase of the field is important; for example, the phase term in Equation (10) is a significant factor for the large variation in Figure 13(e,f).
- For quantifying the contaminant, *C*_X_ can only be determined if coefficients *a*(*λ*), *b*(*λ*), *a*’(*λ*), *b*’(*λ*) are known. A possible strategy is to collect as much scattered light as possible to average out all spectral variations, and if the spatially-averaged *a*(*λ*), *b*(*λ*) are nearly independent of the wavelength, then *a*’(*λ*), *b*’(*λ*) can be omitted, greatly reducing the uncertainty. Suppose there are *L* ≥ 2 wavelengths for measurement, then linear regression can be used to infer the *C*_X_ and coefficient products as discussed in the Appendix Section C, Equations (A.30) and (A.31).

Specifically in regard to the points in (*iv*) and (*v*) above about diversifying and expanding the collection of scattered light, the next section discusses a multispectral imaging result that underscore this consideration.

### 6.4. Near-IR spectral imaging of drug pills

**Figure 14.**
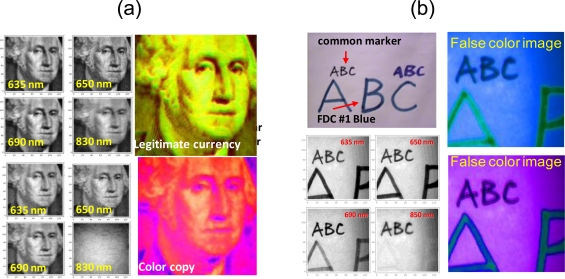
Example of spectral images with features that allow strong discrimination [27]. (a) Legitimate US currency (top left 4 images) *vs.* its color photocopy (bottom left 4 images). The false color images (FCI) right top and bottom are discriminated mainly on the 830 nm spectral images. (b) Similar experiments on common marker dark blue ink *vs*. FDC#1 blue. The two FCI’s show their spectral difference and detailed variation within the large letters.

Conventional spectral imaging is most interesting and performs best when there is a large spectral variation. As an example, Figure 14 shows results of vis-near-IR laser spectral imaging of a US currency banknote, its color photocopy, and some ink drawing on a paper. It is evident that some wavelength such as near-IR 0.83 μm provided large spectral contrast and the discrimination was quite easy and straightforward.

**Figure 15.**
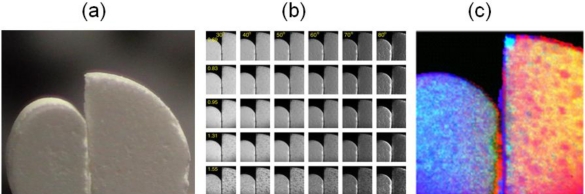
(a) A conventional visible photograph of two drug pills. (b) Scattering coefficient images for wavelength from 0.69 from 1.55 μm (vertical, column) and for viewing angle from 30–80 degree (horizontal, row). (c) False color image of scattering properties of the two pills, taking into account both angular and wavelength data.

However, an example of a more challenging problem, which also underscores the usefulness of laser spectral imaging is the case of drug pills shown in Figure 15. Figure 15(a) shows a conventional visible image (photograph) of two common drug pills, both appeared white. The question is whether there is any spectroscopic feature or difference between them, and what laser spectral imaging can detect. Unlike the glucose problem in Section 6.2, in which the spectral signature is known and the experiment was designed to search for it, there is no prior knowledge of the two pills spectroscopic properties. The experiments were performed for the vis-near IR region, from 0.69 to 1.55 μm. The problem is that they appear to have very little spectroscopic characteristics in this spectral region, and thus pose a more interesting test to laser multispectral imaging.

The 5 × 6 matrix of scattering coefficient **S**(*λ;θ*;**r**) images for wavelength *λ* from 0.69 to 1.55 μm (vertical, column) and for polar scattering angle *θ* from 30 to 80 degree off incident (horizontal, row) are shown in Figure 15(b). In principle, scattering images *vs.* azimuthal angle should have also been measured; however, observation indicated that the azimuthal scattering was generally uniform except for some extreme locations with sharp edges or pointed depression and protrusion. It was decided that these locations were not significant and errors from them were acceptable.

Conventionally, the spectral images of Figure 15(b), in which each pixel is a vector in 30-D space, are processed with various image processing algorithms to enhance the features of interest if they are known or expected. Alternatively, the images can be phenomenologically classified into clusters in the 30-D space. Then three processed images with the most interest aspects can be combined for a RGB FCI. In this case, there is a large amount of information in the 30 images; and the classification can range from very fine to very coarse as desired. Not every piece of information is of interest however. In fact, every single pixel is virtually unique in this 30-D space and can be statistically discriminated from all others. The objective here is not to discriminate various pixels for the sake of discrimination. It is only to compare the pills and see if there is meaningful spectroscopic information. Therefore, the approach is not to interpret the images with phenomenological pattern recognition, but with light scattering principles. For example, simple shadowing effect caused one pill to be spectrally darker than the other. Pure phenomenological classification can claim this as a discriminating feature, but clearly, it is not meaningful for this purpose.

The interpretation must deal with both angular and wavelength-dependence variations in the images, which were far from uniform within the measurement uncertainty over the pill surfaces. Although there was some evidence of spectral signature with long wavelength, 1.31 and 1.55 μm, the amplitude of these signals is only comparable to other variation that is caused by the surface geometrical or morphological effect. As discussed in Section 6.3 above, there is a spectral effect on **S**(**Ω̂***_I_*; **Ω̂***_S_*) of Equation (9) that is unrelated to the spectroscopy of *ε*(*λ*;**r**). In this particular case, it is the geometrical effect associated with *λ*-dependence interference and diffraction. For a random surface, this often gives rise to the speckle effect in high coherence case. Even for incoherent radiation, this effect exists, which is the reason why a surface that is rough in the visible, can act like a mirror in the IR. Some aspect of this effect has been studied and discussed in WMS imaging [22].

The issue here is that the surfaces of the pills were not homogeneous, and the complex *λ*-dependence geometrical effects can be confused with genuine spectroscopic effects. There are macroscopic patches (macroscopic relative to the wavelength) that have different morphologies with different levels of roughness. There are large geometrical structures (100 s of μm in size) such as pockmarks and bumps; all are heterogeneously and randomly distributed. An analogy (but not identical) is the orange peel morphology. Human with prior experience can infer an intensity pattern as a crater or a bump or a rough patch based on an observation of the shadow or shading pattern. However, such interpretation is only an inference based on the human visual learnt experience (implicitly Bayesian), but cannot be rigorously proven whether it is due to spectroscopic effects, e.g., absorption, or to geometrical scattering effects. The net result is that it was difficult to interpret the small intensity variation in the 30 images of Figure 14(b) as something of strictly spectroscopic nature, or associated with the morphological effect of heterogeneous surfaces.

One approach is to determine the net absorption. A model was developed to fit for **S**(*λ;θ*;**r**) as:

(14) $$S(\lambda ;\theta ;r)=a\left[{\left\{\lambda_{i}\right\}}_{i=1}^{L};r\right]\times F\left[\theta ;{\left\{q_{j}(\lambda_{i};r)\right\}}_{j=1}^{r}\right]$$

where $F\left[\theta ;{\left\{q_{j}\left(\lambda_{i};r\right)\right\}}_{j=1}^{r}\right]$ is a phenomenological scattering function that differs from common BSDF via the presence of optics-based *λ*-dependence coefficients ${\left\{q_{j}\left(\lambda_{i};r\right)\right\}}_{j=1}^{r}$. The FDTD calculation of light scattering (Figure 10 is an example) was generated for a range of surface characteristics to provide approximate models to introduce the *λ*-dependence effect for fitting in Equation (14). The goal of the FDTD calculation is not to match to the observed **S**(*λ;θ*;**r**), which would be difficult and unnecessarily labor-intensive. Rather, it is only to provide parameterized models with a rational physical basis for λ-dependence. The parameterized models were used to interpolate and fit to actual **S**(*λ;θ*;**r**), using $a\left[{\left\{\lambda_{i}\right\}}_{i=1}^{L};r\right]$ and ${\left\{q_{j}\left(\lambda_{i};r\right)\right\}}_{j=1}^{r}$ as fitting parameters. The function $F\left[\theta ;{\left\{q_{j}\left(\lambda_{i};r\right)\right\}}_{j=1}^{r}\right]$ is normalized such that:

(15) $$\iint F\left[\theta ;{\left\{q_{j}(\lambda_{i};r)\right\}}_{j=1}^{r}\right]\text{sin}\;\theta d\;\theta d\;\phi =1$$

It is clear that coefficient $a\left[{\left\{\lambda_{i}\right\}}_{i=1}^{L};r\right]$ can be interpreted as the fractional absorption loss according to Equation (9). The advantage of $a\left[{\left\{\lambda_{i}\right\}}_{i=1}^{L};r\right]$ is that the microscopic and macroscopic geometrical effects are no longer dominant.

By mapping $a\left[{\left\{\lambda_{i}\right\}}_{i=1}^{L};r\right]$ for position **r**, an FCI is obtained as in Figure 15(c). The salient features of this result are:

- The two pills are distinguishable as represented with two different colors. The FCI represents a projection of the 5-D wavelength space of all pixels $a\left[{\left\{\lambda_{i}\right\}}_{i=1}^{L};r\right]$ onto a certain 3-D plane for RGB combination that best distinguishes the two pills. Unlike the case of Figure 14 in which a single wavelength, 830 nm serves as the discriminating feature, this 3-D plane projection involves all 5 wavelengths, and is not dominated by any single wavelength, although some wavelengths have more discriminant power than others. In other words, it was not sufficient to project the 5-D pixel clusters onto any plane parallel to any single wavelength axis to distinguish the two pills.
  This simple result is highly deceptive for its apparent obviousness. If the pixels were segmented phenomenologically in the 30-D space and projected into other arbitrary 3-D plane, there would be pixels from both pills grouped together as being in the same category, and spatially adjacent pixels could have been segregated into different categories. The result would have been two pills with mixture of multi-colors pixels, but not necessarily distinguishable from each other except for the overall shape. Here, the two pills are recognized as of slightly different spectroscopic property that is independent of their morphological scattering property.
- The dark spots in the FCI of each pill represent true spectral absorbance that is distinguishable from their surroundings. The main discrimination features here are the l.31 and 1.55 μm as mentioned above. The dark spots were proved not to be results of some intensity variation associated with surface texture variation. And likewise, the effect of surface coarse texture was also removed in the image. In other words, without the dark spots, each pill would have appeared almost of a uniform color.

We hypothesize the dark spots as indicators of pharmaceutical ingredients. Note that their grain size as well as spectral nature is clearly different for the two drug pills. Again, this result is also deceptive for its simplicity. A purely data driven phenomenological classification would form a matrix of both spectral and morphological spots, and it would be difficult to discern the pharmaceutical ingredient particles from the surface bumps and troughs. It is also clear that there are some errors at the sharp edges that had strong scattering and for which the model of Equation (15) was not valid, resulting in wrong classification.

It should be note that the FCI image Figure 15(c) is not a direct construction from various linear combinations of the **S**(*λ;θ*;**r**) of Figure 15(b), but only represents a mapping of coefficient $a\left[{\left\{\lambda_{i}\right\}}_{i=1}^{L};r\right]$, which is fundamentally of a different nature than the conventional scattered light intensity images in Figure 15(b). This result shows the versatility of spectral imaging, which can be applied beyond the original concept of scattered light mapping to generate secondary images (derived images) for relevant information. The difference between the spectral imaging result in this section *vs.* that of Section 5 is that the Section 5 images were obtained with much lower SNR, with lower laser power and at a standoff distance. In compensation, the use of M/LWIR helped better spectroscopic discrimination for those targets in Section 5. In this section, the vis-near-IR imaging of the drug pills enjoyed no such natural spectroscopic contrast. On the other hand, the experiments had much higher SNR. This illustrates the laser advantages of radiometric accuracy and dynamic range that allow the distinction of small spectral difference, which could otherwise be difficult with the passive methods.

## 7. Results on WMS Imaging with Tunable MWIR Lasers

In the results of Section 5 and 6, multi-spectral imaging was applied for spectral discrimination of various targets, but not for the identification of any target with prior spectroscopic knowledge, except for the glucose detection. A multi-spectral imaging system can also perform spectroscopic identification under appropriate condition. This section describes the use of a tunable MWIR laser coupled into the same imaging system to performed WMS absorption imaging of CO gas. More details can be found in [22]. The use of semiconductor-laser-based system for WMS methane gas detection has been well developed and commercialized [33–35]. A fortuitous advantage in this case is the methane overtone absorption line at 1.651 μm, which is within the range of affordable DFB lasers of telecom technology.

**Figure 16.**
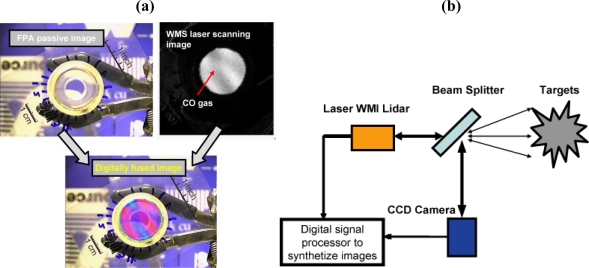
(a) Top left: a CCD visible image of the CO gas tube. Top right: the 2^nd^ order wavelength modulation spectroscopy (WMS) image of the entire scene, measured at the 4.88693 μm, showing only the signature of CO absorption, since all other objects have negligible 2^nd^ order WMS signals. Bottom: a synthetic image, obtained by digital combination of the CCD visible image in top left with the 2^nd^ order WMS image in top right. (b) Concept diagram of a combined passive and active imaging system that would allow synthetic images such as the bottom of (a) [22].

In this experiment, the CO gas was confined to a tube as shown in the top left photograph of Figure 16(a), owing to its toxicity. The transmitted beam was scattered off from a topographic target and detected. The MWIR laser was tuned to the CO absorption line at 4.88693 μm, and the 2^nd^ order WMS measurement was performed. The 2^nd^ order WMS image was shown in the top right of Figure 16(a). Since all other objects in the scene besides CO gas have very small 2^nd^ order derivatives at this particular wavelength, they do not appear on the WMS image, and the result is specific to CO gas detection and identification.

A concept for more practical application is illustrated in Figure 16(b). A synthetic image obtained by digital fusion of a CCD camera image of the gas cell with the 2^nd^ order WMS image of CO is shown in the bottom of Figure 16(a). The fusion was performed on a computer; however, the algorithm can easily be implemented with a dedicated FPGA. More generally, a system with many wavelengths can simultaneously detect different spectroscopic signatures and use color-coding to show different species.

A point worth noting is that imaging also offers additional knowledge, as opposed to spectroscopic sensing of a single point. Although most objects apart from CO gas have very small WMS signature, it is nevertheless detectable above the noise level. This is not the system bias which is a non-zero “baseline” that must be removed as a part of system calibration. In fact, wavelength-modulation imaging (WMI) is more general than WMS and not just for gas detection. The geometrical effects discussed in Section 6 above also have WMI signature [22], which can obfuscate the spectroscopic signature. Imaging allows spatial discrimination, which is the comparison of different points of the scene to decide if the signal is from a background or something that is standout among other objects. Together with multispectral capability, which allows measuring with different wavelengths to improve the specificity with respect to a gas, the technique can overcome these issues to provide more accurate spectroscopic imaging.

## 8. Conclusions

Multi-spectral laser imaging technique using semiconductor lasers was studied and demonstrated for a variety of targets to evaluate the technique capability and potential applications. Semiconductor lasers are significant with regard to potential practicality and affordability; however, they also warrant considerations on system design and engineering. The basic system design, issues on engineering, and measuring approaches are discussed with regard to the tradeoff between the staring imaging technique and scanning imaging technique that is appropriate for low power such as semiconductor lasers.

Although the number of lasers and wavelengths are modest for the system studied, which included a four M/LWIR-wavelength system and five vis-near-IR-wavelength system, the various results in Sections 5–7 show the potential and capability of this technology. For a variety of targets, ranging from common natural and man-made materials, the M/LWIR results demonstrated high multi-spectral resolution to help discriminate complex targets. The issue of diffuse scattering in spectral imaging was also discussed, with examples on glucose measurements and vis-near-IR imaging of drug pills. WMS imaging for gas detection also show its compatibility with conventional spectroscopy.

Multispectral and hyperspectral imaging with passive techniques have been well developed for numerous applications involving target spectral segmentation, discrimination, and identification. Yet, the passive technique cannot avoid some uncertainty and ambiguity on the background radiation, as well as a lack of strong signals in some cases. This is not an issue with laser spectral imaging, as the laser light can be discriminated from the background. Furthermore, although this works involved only elastic scattering imaging, the system design and analysis here are obviously relevant and applicable to other laser spectroscopic techniques such as Raman, fluorescence, photoacoustic, or nonlinear optics. As laser spectroscopic sensing is well proven of its value, it is natural to extend the laser spectroscopic capability into the multi-spectral imaging domain that can offer the combined power and capability of both techniques. This paper aims to contribute to the development of this trend.

## Figures and Tables

**Figure A.1.**
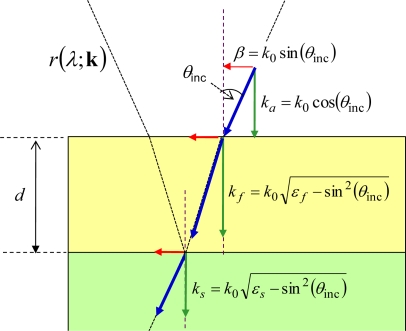
Reflectance from a thin-film layer on a dielectric substrate.

## Appendix

### A. System noise considerations for parallel and sequential measurements

This section considers the calculation of the laser power and image acquisition time for the parallel and sequential methods as discussed in Section 2.3. For the sequential method, the signal-to-noise ratio (SNR) of a single measurement (single pixel, single wavelength) is:

(A.1) $$\mathrm{SNR}=\rho \frac{P\sqrt{\tau }}{\mathrm{NEP}}\;$$

where *P* is the laser power, *ρ* is the fraction of incident power that is returned as the signal, *τ* is the measurement time, and *NEP* is the average noise equivalent power of the receiver. From Equation (A.1), for a given desired SNR, the power required is:

(A.2) $$P=\frac{1}{\rho }\mathrm{SNR}\frac{\mathrm{NEP}}{\sqrt{\tau }}$$

From Equation (A.2), the measurement time required for each pixel for a given power is:

(A.3) $$\tau ={\left(\frac{1}{\rho P}\mathrm{SNR}\;\mathrm{NEP}\right)}^{2}$$

The total time required to complete an image with the sequential method is:

(A.4) $$T_{\mathrm{sequent}}=\mathrm{QL}(\tau +t_{0})=\mathrm{QL}\left[t_{0}+{\left(\frac{1}{\mathrm{\rho P}}\mathrm{SNR}\;\mathrm{NEP}\right)}^{2}\right]$$

where subscript *sequent* denotes the sequential measurement method, *Q* is the number of spatial pixels, *L* is the number of wavelengths, and *t*_0_ is the dead time when the system is busy, changing wavelength or illuminating spot and does not perform a measurement.

For the parallel method, assume that *P* is simultaneously divided among all pixel-*λ* product *QL*, Then, the average SNR of each pixel is:

(A.5) $$\mathrm{SNR}=\rho \frac{P\sqrt{\tau }}{\mathrm{QL}\;\mathrm{NEP}}$$

and Equations (A.2) and (A.3) now become:

(A.6) $$P=\frac{1}{\rho }\mathrm{QL}\;\mathrm{SNR}\frac{\mathrm{NEP}}{\sqrt{\tau }}$$

(A.7) $$\tau ={\left(\frac{1}{\rho P}\mathrm{QL}\;\mathrm{SNR}\;\mathrm{NEP}\right)}^{2}\equiv T_{\mathrm{paral}}$$

More generally, a hybrid approach can divide *QL* into block *M* × *N*, which means *M* sequential measurements of *N* parallel-measured blocks. Then, the time is:

(A.8) $$T_{M,N}=M\left[t_{0}+{\left(\frac{N}{\rho P}\mathrm{SNR}\;\mathrm{NEP}\right)}^{2}\right]$$

Comparing *T_sequent_* of Equation (A.4) and *T_paral_* ≡ *τ* of Equation (A.7):

(A.9.a) $$\frac{T_{\mathrm{sequent}}}{T_{\mathrm{paral}}}=\frac{\left(1+\eta \right)}{\mathrm{QL}}$$

where, for simplicity *η* is defined:

(A.9.b) $$\eta \equiv t_{0}/{\left(\frac{1}{\rho P}\mathrm{SNR}\;\mathrm{NEP}\right)}^{2}$$

it appears that the sequential method allows faster (more efficient) image acquisition than the parallel method as a function of increasing *QL*. Conversely, for the same total image acquisition time *T*, the power required for the sequential method in Equation (A.2) is less than that for the parallel method in Equation (A.6):

(A.10) $$\frac{P_{\mathrm{sequent}}}{P_{\mathrm{paral}}}=\sqrt{\frac{\left(1+\eta \right)}{\mathrm{QL}}}$$

A more realistic noise model involves also the laser RIN (relative intensity noise). In this case, the term *NEP* in the above equations is replaced by:

(A.11) $$\mathrm{NEP}\to \sqrt{{\mathrm{NEP}}^{2}(f_{s})+{\left[\rho P\;\mathrm{RIN}(f_{s})\right]}^{2}}$$

for the sequential term, and:

(A.12) $$\mathrm{NEP}\to \sqrt{{\mathrm{NEP}}^{2}(F_{p})+{\left[\rho P\;\mathrm{RIN}(F_{p})/QL\right]}^{2}}$$

for the parallel term. They are simply the additive combination of receiver noise *NEP* and laser RIN. For both, explicit frequency-dependence is shown to include realistic noise behaviors, which often involve 1/*f*-component; and:

(A.13.a) $$f_{s}\approx \frac{1}{\tau_{s}}=\frac{{\left(\rho P\right)}^{2}}{{\mathrm{SNR}}^{2}\left({\mathrm{NEP}}^{2}(f_{s})+{\left[\rho P\;\mathrm{RIN}(f_{s})\right]}^{2}\right)}$$

(A.13.b) $$F_{p}\approx \frac{1}{\tau_{p}}=\frac{{\left(\rho P\right)}^{2}}{{\left(\mathrm{SNR}\;\mathrm{QL}\right)}^{2}\left({\mathrm{NEP}}^{2}(F_{p})+{\left[\rho P\;\mathrm{RIN}(F_{p})/\mathrm{QL}\right]}^{2}\right)}$$

which represent the measurement frequencies of the serial and parallel methods, respectively. Certainly, the laser intensity fluctuation effect, *i.e.*, RIN can be reduced by dividing the received signal with the monitored laser transmitted power. However, this can be sometimes impractical with respect to system cost and complexity.

Using Equations (A.11)–(A.13), Equation (A.9.a) becomes:

(A.14) $$\frac{T_{\mathrm{sequent}}}{T_{\mathrm{paral}}}=\frac{\left(1+\eta \right)}{\mathrm{QL}}\times \frac{{\mathrm{NEP}}^{2}(f_{s})+{\left[\rho P\;\mathrm{RIN}(f_{s})\right]}^{2}}{{\mathrm{NEP}}^{2}(F_{p})+{\left[\rho P\;\mathrm{RIN}(F_{p})/\mathrm{QL}\right]}^{2}}$$

which asymptotically approaches to Equation (A.9.a) if laser RIN is negligible compared to the receiver noise. Both Equations (A.13.a) and (A.13.b) entail a relationship, as *f_s_* and *F_p_* appear on both sides of the equations. This reflects the case when the noise power spectral density is not a flat white spectrum, but may have *f*-dependency. It means that the desire SNR is achievable only if there is a solution for (A.13.a) and (A.13.b).

### B. Reflection from a thin-film on a dielectric substrate

This section derives Equations (10) and (11) in Section 6.2. Consider the case in Figure A.1, which is a thin-film of material with dielectric function *ε_f_*(*λ*) on a substrate with dielectric function *ε_s_*(*λ*).

The reflection coefficient of a plane wave with wave vector **k** and incident angle θ_inc_ can be obtained by imposing the continuity boundary condition for the **E** and **H** field in the *y*–*z* plane at each interface. For the TE mode, *E_y_* and *H_z_* for each region, air, film, substrate are:

(A.15.a) $$\text{Air}:\;\;\;E_{y}=E_{0}\;e^{i(\beta z-\omega t)}\left(\begin{array}{cc} e^{ik_{a}x} & e^{-ik_{a}x} \end{array}\right)\cdot \left(\begin{array}{c} 1\\ r \end{array}\right);\;\;\;H_{z}=-\frac{iE_{0}}{\mu k_{0}}e^{i(\beta z-\omega t)}\left(\begin{array}{cc} ik_{a}e^{ik_{a}x} & -ik_{a}e^{-ik_{a}x} \end{array}\right)\cdot \left(\begin{array}{c} 1\\ r \end{array}\right)$$

(A.15.b) $$\begin{array}{ll} \text{Flim}: & E_{y}=E_{0}\;e^{i(\beta z-\omega t)}\;\left(\begin{array}{cc} e^{ik_{f}\;x} & e^{-ik_{f}\;x} \end{array}\right)\cdot \left(\begin{array}{c} c_{1}\\ c_{2} \end{array}\right)\\ & H_{z}=-\frac{iE_{0}}{\mu k_{0}}\;e^{i(\beta z-\omega t)}\;\left(\begin{array}{cc} ik_{f}\;e^{ik_{f}\;x} & -ik_{f}\;e^{-ik_{f}\;x} \end{array}\right)\cdot \left(\begin{array}{c} c_{1}\\ c_{2} \end{array}\right) \end{array}$$

(A.15.c) $$\begin{array}{ll} \text{Substrate}: & E_{y}=E_{0}\;e^{i(\beta z-\omega t)}\;\left(\begin{array}{cc} e^{ik_{s}\;(x-d)} & e^{-ik_{s}\;(x-d)} \end{array}\right)\cdot \left(\begin{array}{c} t\\ 0 \end{array}\right);\\ & H_{z}=-\frac{iE_{0}}{\mu k_{0}}\;e^{i(\beta z-\omega t)}\;\left(\begin{array}{cc} ik_{s}\;e^{ik_{s}\;(x-d)} & -ik_{s}\;e^{-ik_{s}\;(x-d)} \end{array}\right)\cdot \left(\begin{array}{c} t\\ 0 \end{array}\right) \end{array}$$

where *k*_0_ ≡ 2*π/λ*, *μ* is the magnetic permeability, which is the same for all layer in this case, *ω* is the light frequency, and other quantities are defined in Figure A.1. Two relevant coefficients are reflection *r* in Equation (A.15.a) and transmission *t* in Equation (A.15.c); coefficients *c*_1_ and *c*_2_ in Equation (A.15.c) are simply some values to be solved by imposing the boundary condition.

It is convenient to put Equations (A.15.a, b, c) in the matrix form, and drop term *E*_0_*e*^*i*(*βz*–ω*t*)^ that is common to all; then the continuity of *E_y_* and *H_z_* at each interface gives:

(A.16.a) $$\text{Air-film}:\;\;\;\;\;\;\;\;\;\;\;{\left(\begin{array}{c} E_{y}\\ i\mu k_{0}H_{z} \end{array}\right)|}_{x=0}=\left(\begin{array}{cc} 1 & 1\\ ik_{a} & -ik_{a} \end{array}\right)\cdot \left(\begin{array}{c} 1\\ r \end{array}\right)=\left(\begin{array}{cc} 1 & 1\\ ik_{f} & -ik_{f} \end{array}\right)\cdot \left(\begin{array}{c} c_{1}\\ c_{2} \end{array}\right)$$

(A.16.b) $$\text{Film-substrate}:\;\;\;\;\;\;\;\;{\left(\begin{array}{c} E_{y}\\ i\mu k_{0}H_{z} \end{array}\right)|}_{x=d}=\left(\begin{array}{cc} e^{ik_{f}\;d} & e^{-ik_{f}\;d}\\ ik_{f}\;e^{ik_{f}\;d} & -ik_{f}\;e^{-ik_{f}\;d} \end{array}\right)\cdot \left(\begin{array}{c} c_{1}\\ c_{2} \end{array}\right)=\left(\begin{array}{cc} 1 & 1\\ ik_{s} & -ik_{s} \end{array}\right)\cdot \left(\begin{array}{c} t\\ 0 \end{array}\right)$$

Eliminating coefficients *c*_1_ and *c*_2_ in both Equations (A.16.a and b) yields:

(A.17) $$\left(\begin{array}{c} 1\\ r \end{array}\right)={\left(\begin{array}{cc} 1 & 1\\ ik_{a} & -ik_{a} \end{array}\right)}^{-1}\left(\begin{array}{cc} 1 & 1\\ ik_{f} & -ik_{f} \end{array}\right)\cdot {\left(\begin{array}{cc} e^{ik_{f}\;d} & e^{-ik_{f}\;d}\\ ik_{f}\;e^{ik_{f}\;d} & -ik_{f}\;e^{-ik_{f}\;d} \end{array}\right)}^{-1}\left(\begin{array}{cc} 1 & 1\\ ik_{s} & -ik_{s} \end{array}\right)\cdot \left(\begin{array}{c} t\\ 0 \end{array}\right)$$

which can be solved to give:

(A.18.a) $$r_{\text{TE}}(\lambda ;k)=\frac{\left(k_{a}-k_{f})(k_{f}+k_{s})+e^{2\mathrm{id}k_{f}}\;(k_{a}+k_{f})(k_{f}-k_{s}\right)}{\left(k_{a}+k_{f})(k_{f}+k_{s})+e^{2\mathrm{id}k_{f}}\;(k_{a}-k_{f})(k_{f}-k_{s}\right)}$$

(A.18.b) $$t_{\text{TE}}(\lambda ;k)=\frac{4k_{a}k_{f}\;e^{\mathrm{id}k_{f}}}{\left(k_{a}+k_{f})(k_{f}+k_{s})+e^{2\mathrm{id}k_{f}}\;(k_{a}-k_{f})(k_{f}-k_{s}\right)}$$

where subscript TE indicates transverse electric. A similar result can be obtained for the TM mode:

(A.19) $$\begin{array}{l} \left(\begin{array}{c} 1\\ r \end{array}\right)={\left(\begin{array}{cc} 1 & 1\\ ik_{a} & -ik_{a} \end{array}\right)}^{-1}\left(\begin{array}{cc} 1 & 0\\ 0 & \frac{1}{\varepsilon_{f}} \end{array}\right)\left(\begin{array}{cc} 1 & 1\\ ik_{f} & -ik_{f} \end{array}\right)\cdot \\ \;\;\;\;\;\;{\left(\begin{array}{cc} e^{ik_{f}\;d} & e^{-ik_{f}\;d}\\ ik_{f}\;e^{ik_{f}\;d} & -ik_{f}\;e^{-ik_{f}\;d} \end{array}\right)}^{-1}\left(\begin{array}{cc} 1 & 0\\ 0 & \frac{\varepsilon_{f}}{\varepsilon_{s}} \end{array}\right)\left(\begin{array}{cc} 1 & 1\\ ik_{s} & -ik_{s} \end{array}\right)\cdot \left(\begin{array}{c} t\\ 0 \end{array}\right) \end{array}$$

which yields:

(A.20.a) $$r_{\text{TM}}(\lambda ;k)=\frac{\left(\varepsilon_{f}\;k_{a}-k_{f})(\varepsilon_{s}\;k_{f}+\varepsilon_{f}\;k_{s})+e^{2\mathrm{id}k_{f}}\;(\varepsilon_{f}\;k_{a}+k_{f})(\varepsilon_{s}\;k_{f}-\varepsilon_{f}\;k_{s}\right)}{\left(\varepsilon_{f}\;k_{a}+k_{f})(\varepsilon_{s}\;k_{f}+\varepsilon_{f}\;k_{s})+e^{2\mathrm{id}k_{f}}\;(\varepsilon_{f}\;k_{a}-k_{f})(\varepsilon_{s}\;k_{f}-\varepsilon_{f}\;k_{s}\right)}$$

(A.20.b) $$t_{\text{TM}}(\lambda ;k)=\frac{4k_{a}\;k_{f}\;\varepsilon_{s}\;\varepsilon_{f}\;e^{\mathrm{id}k_{f}}}{\left(\varepsilon_{f}\;k_{a}+k_{f})(\varepsilon_{s}\;k_{f}+\varepsilon_{f}\;k_{s})+e^{2\mathrm{id}k_{f}}\;(\varepsilon_{f}\;k_{a}-k_{f})(\varepsilon_{s}\;k_{f}-\varepsilon_{f}\;k_{s}\right)}$$

These results are used to plot Figure 13(e,f).

In the glucose experiments described in Section 6.2, the beam is not a plane wave, but a focused beam. Thus, the beam can be expressed as a linear superposition of plane waves:

(A.21) $$E(r)=\int A(k)e^{i\phi (k)}e^{ik\cdot r}dk$$

Each **k** component of Equation (A.21) contributes a reflection wave with amplitude:

(A.22) $$r(\lambda ;k)A(k)e^{i\phi (k)}$$

where *r*(*λ*;**k**) is given in Equation (A.18.a) or (A.20.a), depending on the polarization. The reflected beam is a coherent sum of all reflected waves of Equation (A.22):

(A.23) $$E_{\text{refl}}(r)=\int r(\lambda ;k)A(k)e^{i\phi (k)}e^{ik_{\text{refl}}\cdot r}dk$$

where $k_{\text{refl}}=(\begin{array}{lll} -k_{a} & \beta_{y} & \beta_{z} \end{array})$, where *β_y_*, *β_z_* are the incident wave vector components in the *y*–*z* plane. Since the beam has low NA focusing, with little transverse spatial component, one can approximate *e*^*i***k**_refl_·**r**^ as a constant *e*^*i***k**_0_·**r**^, which can be taken out of the integral. Then the collected power of the scattered beam is:

(A.24) $$S({\hat{\Omega }}_{S})\equiv R_{T}\;(\lambda )={\left|\iint_{\begin{array}{l} \text{Receiver}\\ \text{aperture} \end{array}}H({\hat{\Omega }}_{S})d{\hat{\Omega }}_{S}r(\lambda ;k)A(k)e^{i\phi (k)}dk\right|}^{2}$$

where *H*(**Ω̂***_S_*) is the receiver collection efficiency for beam in the viewing (receiving) angle **Ω̂***_S_*, and the integration is over the receiver angular aperture. If the receive responsivity is uniform over its field of view, *H*(**Ω̂***_S_*) is simply = 1.

It should be noted that Equation (A.24) has general validity for diffuse scattering and is not limited to that of Figure (A.1); and it was used for the FDTD computer simulation of scattering from a rough surface as illustrated in Figure 10, except that coefficient *r*(*λ*;**k**) is not that of Equations (A.18.a) or (A.20.a) but represents the backscattering amplitude.

### C. Detection of small contamination in elastic diffuse scattering:

Consider the case of scattering illustrated in Figure 11(b), but with the generalization for any solvent with a small contamination. Let the film have a known dielectric function *ε_f_*(*λ*), which is altered to become *ε_f_*(*λ;C*_X_) because of some contaminant X, and *C*_X_ is its concentration. It is expected that |[*ε_f_*(*λ;C*_X_)–*ε_f_*(*λ*)]/*ε_f_*(*λ*)| is small, such as ∼10^−3^ for glucose in Section 6.2 and even smaller in other cases. The question is what is the physical basis for the contamination to be detected and quantified via elastic scattering? In other words, how does *C*_X_ manifest itself in the scattered light, and how does one detect and quantify *C*_X_.

One can generally assume that the scattered electric field amplitude **E***_S_* is an unknown but deterministic function of *ε_f_*(*λ;C*_X_) and substrate dielectric function *ε_s_*(*λ*). The scattered electric field **E***_S_*[*λ;ε_s_*(*λ*),*ε_f_*(*λ;C*_X_)] can be expanded with the first-order Taylor’s series:

(A.25) $$E_{S}\;\left[\lambda ;\varepsilon_{s}\;(\lambda ),\varepsilon_{f}\;(\lambda ;C_{X})\right]\approx E_{S}\;\left[\lambda ;\varepsilon_{s}\;(\lambda ),\varepsilon_{f}\;(\lambda ;0)\right]+C_{X}\;\frac{\partial E_{S}}{\partial \varepsilon_{f}}{\tilde{\varepsilon }}_{X}$$

where *ε̃*_X_ ≡ *ε̂*_X_ – *με_f_*,, and *ε̂*_X_ is the specific dielectric function per unit of substance concentration, and *μ* is a substitution factor to account for the displacement of solvent molecules by those of substance X. It should be noted that the model of Equation (A.25) is generic and not limited to the configuration in Figure 11(b). The detected scattered field is a sum of all those in Equation (A.25), and the total collected scattered intensity *S* is:

(A.26.a) $$S\;[\lambda ;\varepsilon_{s}\;(\lambda ),\;\varepsilon_{f}\;(\lambda ;C_{X})]=|U{|}^{2}$$

where:

(A.26.b) $$U=\int E_{S;k}\;dk+C_{X}{\tilde{\varepsilon }}_{X}\int \frac{\partial E_{S;k}}{\partial \varepsilon_{f}}dk\equiv U_{0}+C_{X}{\tilde{\varepsilon }}_{X}\frac{\partial U_{0}}{\partial \varepsilon_{f}}$$

Applying Equation (A.26.b) in Equation (A.26.a), and keep the 1^st^ order of *C*_X_, and furthermore, split *ε̃*_X_ into its Re and Im component, then *S* consists of a background term without *C*_X_ and a term with *C*_X_:

(A.27) $$S\left[\lambda ;\varepsilon_{s}\;(\lambda ),\varepsilon_{f}\;(\lambda ;C_{X})\right]=S_{0}+2C_{X}\;\left\{\text{Re}\;{\tilde{\varepsilon }}_{X}\;\text{Re}\left[U•\frac{\partial U^{*}}{\partial \varepsilon_{f}}\right]+\text{Im}{\tilde{\varepsilon }}_{X}\;\text{Im}\left[U•\frac{\partial U^{*}}{\partial \varepsilon_{f}}\right]\right\}$$

where *S*_0_ = |**U**_0_|^2^. For the derivative spectrum in the manner of WMS, the 1^st^ order is:

(A.28.a) $$\frac{d}{d\lambda }S\left[\lambda ;\varepsilon_{s}\;(\lambda ),\varepsilon_{f}\;(\lambda ;C_{X})\right]=\frac{dS_{0}}{d\lambda }+2C_{X}\;\left(\begin{array}{cccc} \text{Re}\;{\tilde{\varepsilon }}_{X} & \text{Im}\;{\tilde{\varepsilon }}_{X} & \frac{d\;\text{Re}\;{\tilde{\varepsilon }}_{X}}{d\lambda } & \frac{d\;\text{Im}\;{\tilde{\varepsilon }}_{X}}{d\lambda } \end{array}\right)•\left(\begin{array}{c} \frac{\mathrm{da}}{d\lambda }\\ \frac{\mathrm{db}}{d\lambda }\\ a\\ b \end{array}\right)$$

where:

(A.28.b) $$a+ib\equiv U•\frac{\partial U^{*}}{\partial \varepsilon_{f}}\left(\int E_{S;k}\left[\lambda ;\varepsilon_{S}\;(\lambda ),\varepsilon_{f}\;(\lambda )\right]dk\right)•\left(\int \frac{\partial }{\partial \varepsilon_{f}}E_{s;k^{\prime }}^{*}\left[\lambda ;\varepsilon_{s}\;(\lambda ),\varepsilon_{f}\;(\lambda )\right]dk\right)$$

Also:

(A.28.c) $$\frac{dS_{0}}{d\lambda }\equiv \frac{d}{d\lambda }S\left[\lambda ;\varepsilon_{s}\;(\lambda ),\varepsilon_{f}\;(\lambda )\right]=\frac{\partial S}{\partial \lambda }+\frac{\partial S}{\partial \varepsilon_{s}}\frac{d\varepsilon_{s}}{d\lambda }+\frac{\partial S}{\partial \varepsilon_{f}}\frac{d\varepsilon_{f}}{d\lambda }$$

In light of Equations (A.28), any *C*_X_ -dependent result in Figure 13 can be interpreted as a linear combination of various *ε̃*_X_ terms in (A.28.a) column vector. The following are can be inferred:

- It is desirable that the term *dS*_0_/*dλ* of Equation (A.28.c) does not have spectral features that mask those of Re*ε̃*_X_(*λ*), Im*ε̃*_X_(*λ*), *d* Re *ε̃*_X_/*dλ*, and *d* Im *ε̃*_X_/*dλ* of Equation (A.28.a). For the glucose experiments in Section 6.2, neither water nor the substrate has sharp features in the 9–10 μm range. *dε_s_*/*dλ* and *dε_f_*/*dλ* contribute at most a gradual linear baseline, which does not overwhelm *d* Re*ε̃*_X_/*dλ* and *d* Im *ε̃*_X_/*dλ*. It is obviously futile to detect the signature of contaminant X over a spectral range in which the solvent and/or a substrate have the same or similar spectral signature.
- It is desirable that coefficients *a*(*λ*), *b*(*λ*), *a*’(*λ*), *b*’(*λ*) be as large as possible. In Equation (A.28.b), the magnitude of *a*(*λ*) and *b*(*λ*) depends on two terms. Normalized to incident laser power, the crucial term determining the coefficient magnitude is:
  (A.29) $$\left|\frac{\frac{\partial }{\partial \varepsilon_{f}}\int E_{S;k^{\prime }}\left[\lambda ;\varepsilon_{s}\;(\lambda ),\varepsilon_{f}\;(\lambda )\right]dk}{\int E_{S;k}\left[\lambda ;\varepsilon_{s}\;(\lambda ),\varepsilon_{f}\;(\lambda )\right]dk}\right|$$
  This term entails that it is most desirable for the scattered field to be sensitive to the film dielectric constant. This is also intuitively self-explanatory, since the measurement aims to detect very small modification to the film *ε_f_*(*λ*), hence the scattered field has to be insensitive to this quantity to detect a small change.
- With respect to the problem of quantification, *C*_X_ cannot be determined independently unless the coefficients are known. A desirable circumstance is that *a’(λ*), *b’(λ*) are negligible as discussed in Section 6.3. Suppose there are *L* ≥ 2 wavelengths for measurement, then linear regression can be used to infer the *C*_X_ and coefficient products from the second-term of the right hand side of Equation (A.28.a):
  (A.30) $$2\left(\begin{array}{cc} \text{Re}\;{{\tilde{\varepsilon }}^{\prime }}_{X}\;(\lambda_{1}) & \text{Im}\;{{\tilde{\varepsilon }}^{\prime }}_{X}\;(\lambda_{1})\\ \text{Re}\;{{\tilde{\varepsilon }}^{\prime }}_{X}\;(\lambda_{2}) & \text{Im}\;{{\tilde{\varepsilon }}^{\prime }}_{X}\;(\lambda_{2})\\ \vdots & \vdots \\ \text{Re}\;{{\tilde{\varepsilon }}^{\prime }}_{X}\;(\lambda_{L}) & \text{Im}\;{{\tilde{\varepsilon }}^{\prime }}_{X}\;(\lambda_{L}) \end{array}\right)•\left(\begin{array}{c} C_{X}a\\ C_{X}b \end{array}\right)=\left(\begin{array}{c} \mathrm{dS}(\lambda_{1})/d\lambda \\ \mathrm{dS}(\lambda_{2})/d\lambda \\ \vdots \\ \mathrm{dS}(\lambda_{L})/d\lambda \end{array}\right)$$

There are additional possible strategies to isolate the coefficients from *C*_X_, for example, the coefficients also appear in the term (*∂S*/*∂ε_f_*)(*dε_f_*/*dλ*) of Equation (A.28.c), and a similar equation can be obtained:

(A.31) $$2\left(\begin{array}{cc} \text{Re}\;d\;\varepsilon_{f}\;(\lambda_{1}{}^{\prime })/d\lambda & \text{Im}\;d\;\varepsilon_{f}\;(\lambda_{1}{}^{\prime })/d\lambda \\ \text{Re}\;d\;\varepsilon_{f}\;(\lambda_{2}{}^{\prime })/d\lambda & \text{Im}\;d\;\varepsilon_{f}\;(\lambda_{2}{}^{\prime })/d\lambda \\ \vdots & \vdots \\ \text{Re}\;d\;\varepsilon_{f}\;(\lambda_{M}{}^{\prime })/\partial \lambda & \text{Im}\;d\;\varepsilon_{f}\;(\lambda_{M}{}^{\prime })/d\lambda \end{array}\right)•\left(\begin{array}{c} a\\ b \end{array}\right)=\left(\begin{array}{c} \mathrm{dS}(\lambda_{1}{}^{\prime })/d\lambda \\ \mathrm{dS}(\lambda_{2}{}^{\prime })/d\lambda \\ \vdots \\ \mathrm{dS}(\lambda_{M}{}^{\prime })/d\lambda \end{array}\right)$$

provided that the wavelength set *λ*_1_’, *λ*_2_’,… *λ_M_*’ is such that Equation (A.30) is negligible and so are other terms in Equation (A.28.c). In essence, Equation (A.31) is to use the film *ε_f_*(*λ*) as the calibration material to determine *a* and *b*, which then can be used subsequently to infer *C*_X_.
